# Stemness analysis in hepatocellular carcinoma identifies an extracellular matrix gene–related signature associated with prognosis and therapy response

**DOI:** 10.3389/fgene.2022.959834

**Published:** 2022-08-30

**Authors:** Lei Chen, Dafang Zhang, Shengmin Zheng, Xinyu Li, Pengji Gao

**Affiliations:** ^1^ Department of Hepatobiliary Surgery, Peking University People’s Hospital, Beijing, China; ^2^ Department of General Surgery, Beijing Jishuitan Hospital, Beijing, China

**Keywords:** hepatocellular carcinoma, stemness, extracellular matrix–related genes, prognosis, tumor microenvironment, therapeutic response

## Abstract

**Background:** Tumor stemness is the stem-like phenotype of cancer cells, as a hallmark for multiple processes in the development of hepatocellular carcinoma (HCC). However, comprehensive functions of the regulators of tumor cell’s stemness in HCC remain unclear.

**Methods:** Gene expression data and clinical information of HCC samples were downloaded from The Cancer Genome Atlas (TCGA) dataset as the training set, and three validation datasets were derived from Gene Expression Omnibus (GEO) and International Cancer Genome Consortium (ICGC). Patients were dichotomized according to median mRNA expression–based stemness index (mRNAsi) scores, and differentially expressed genes were further screened out. Functional enrichment analysis of these DEGs was performed to identify candidate extracellular matrix (ECM)–related genes in key pathways. A prognostic signature was constructed by applying least absolute shrinkage and selection operator (LASSO) to the candidate ECM genes. The Kaplan–Meier curve and receiver operating characteristic (ROC) curve were used to evaluate the prognostic value of the signature. Correlations between signatures and genomic profiles, tumor immune microenvironment, and treatment response were also explored using multiple bioinformatic methods.

**Results:** A prognostic prediction signature was established based on 10 ECM genes, including *TRAPPC4*, *RSU1*, *ILK*, *LAMA1*, *LAMB1*, *FLNC*, *ITGAV*, *AGRN*, *ARHGEF6*, and *LIMS2*, which could effectively distinguish patients with different outcomes in the training and validation sets, showing a good prognostic prediction ability. Across different clinicopathological parameter stratifications, the ECMs signature still retains its robust efficacy in discriminating patient with different outcomes. Based on the risk score, vascular invasion, *α*-fetoprotein (AFP), T stage, and N stage, we further constructed a nomogram (C-index = 0.70; AUCs at 1-, 3-, and 5-year survival = 0.71, 0.75, and 0.78), which is more practical for clinical prognostic risk stratification. The infiltration abundance of macrophages M0, mast cells, and Treg cells was significantly higher in the high-risk group, which also had upregulated levels of immune checkpoints PD-1 and CTLA-4. More importantly, the ECMs signature was able to distinguish patients with superior responses to immunotherapy, transarterial chemoembolization, and sorafenib.

**Conclusion:** In this study, we constructed an ECM signature, which is an independent prognostic biomarker for HCC patients and has a potential guiding role in treatment selection.

## Introduction

Liver cancer (LC) is one of the most prevalent tumors in the world, ranking as the sixth most common cancer and the third cause of cancer-related deaths worldwide (Sung et al., 2021). The World Health Organization estimated that more than 1 million people will die of LC by 2030 (Villanueva, 2019), which indicates that both the incidence and mortality of LC will increase significantly (Bresnahan et al., 2020). It is a group of heterogeneous malignant tumors with different histological features and outcomes, mainly including hepatocellular carcinoma (HCC), intrahepatic cholangiocarcinoma, and mixed hepatocellular cholangiocarcinoma (Sia et al., 2017; Yang et al., 2019). Patients with HCC are usually diagnosed at advanced stages and are not suitable for the resect surgery because of poor prognosis and a high recurrence rate (Forner et al., 2018). At the same time, the molecular mechanism of HCC is complex, usually involving a variety of genetic abnormalities, such as genomic instability, single nucleotide polymorphisms, somatic mutations, and dysregulation of signaling pathways, which are all related to the occurrence and development of HCC (Tang et al., 2021). Although many advances have been made in the treatments in the past decades, the overall survival of HCC patients is still unsatisfied (Zhang et al., 2018). To improve the management of HCC, novel prognosis biomarkers are urgently needed to evaluate the outcomes and therapy efficacy for patients with HCC.

Cancer stem cells (CSCs) are a small group of undifferentiated cells in tumor tissue and can induce unlimited self-renewal of heterogeneous tumor cells (Kreso and Dick, 2014). A previous study has shown that CSCs are not only crucial for tumor growth and maintenance but also associated with tumor recurrence and metastasis (Peitzsch et al., 2013; Nassar and Blanpain, 2016). Cancer stemness has also been considered to play an important role in HCC (Tsui et al., 2020), supported by the increasing events of genomic, epigenomic, transcriptomic, and proteomic alterations related to it (Eppert et al., 2011). It is well known that the high recurrence rate of HCC is partly due to the presence of CSCs. Furthermore, the sensitivity of conventional radiotherapy and chemotherapy is limited attributed by the biological characteristics of CSCs and protective effect of tumor microenvironment (TME) (Yi et al., 2020). mRNA expression–based stemness index (mRNAsi) is a novel quantitative method to describe the similarities between cancer cells and CSCs, and higher mRNAsi scores are correlated with active biological processes in CSCs and greater tumor dedifferentiation, as reflected by histopathological grades (Pan et al., 2019). It has been identified as an effective marker associated with tumor recurrence and treatment resistance (Malta et al., 2018). Meanwhile, TME not only regulates cancer stemness but also cooperates with CSCs to endure multiple stress condition (Carloni et al., 2014). The extracellular matrix (ECM) as a major structural component of the TME is composed mainly of biochemically distinct components such as fibrous proteins, glycoproteins, proteoglycans, and polysaccharides (Poltavets et al., 2018). It has been revealed that ECM plays a crucial role in normal and CSCs as the niche (Kreso and Dick, 2014). According to previous studies, ECM stiffness had been implicated to promote cancer stemness gene expression to drive the tumor-initiation activity of HCC stem cells (You et al., 2016). The ECM–receptor interaction pathway has been demonstrated as the most significant pathway in cancer (Tian et al., 2021). However, the mechanistic implications of mRNAsi on cancer biology are incompletely understood, and no study has previously attempted to identify the prognostic value of ECMs-related genes and their relationship with mRNAsi in HCC.

In this study, we developed a novel ECM-related signature for predicting the prognosis of HCC using data from the TCGA database. Then we validated its prognostic prediction capacity using data from the GEO database and evaluated its correlation with clinicopathological features, genetic alterations, immune microenvironment, and therapeutic response. This signature is expected to provide potential guidance for personalized treatment of HCC patients.

## Materials and methods

### Study design

A schematic workflow of this study is present in Supplementary Figure S1. In short, we utilized The Cancer Genome Atlas Liver Hepatocellular Carcinoma (TCGA-LIHC) dataset as the training set, and, three validation datasets were downloaded from Gene Expression Omnibus (GEO) and International Cancer Genome Consortium (ICGC). First, we dichotomized TCGA-LIHC patients according to the median mRNAsi score and compared the overall survival (OS) and disease-free survival (DFS) between the two groups. Subsequently, we compared the gene expression feature of patients from the two groups to screen out differentially expressed genes (DEGs) and then performed a functional enrichment analysis on them to select key pathways and defined the related candidate genes. Third, a least absolute shrinkage and selection operator (LASSO) regression analysis was performed on these candidate genes, and a prognostic model was constructed using TCGA-LIHC data and validated in the GEO cohorts and the ICGC cohort. Then by combination with the prognostic model and clinical characteristics, a nomogram suitable for clinical application was constructed. Finally, the correlation between genomic profile, TME, therapy response, and risk score were analyzed.

### Data source and acquisition of candidate genes

In total, 355 patients with expression data of HCC were obtained from the TCGA database (https://portal.gdc.cancer.gov/). GSE54236 with 161 samples and ICGC-LIRI-JP with 260 samples were retrieved from the GEO (https://www.ncbi.nlm.nih.gov/geo/) or ICGC (https://dcc.icgc.org/) as independent validation datasets. In addition, transcriptome data and calculated tumor volume doubling times of the GSE54236 cohort were used to explore the relationship between the risk score and rapid tumor growth. The tumor volume doubling time was calculated based on imaging data (Villa et al., 2016). The stemness index data for HCC, including mRNAsi and epigenetically regulated mRNAsi (EREG-mRNAsi) were downloaded from a published study (Supplementary Table S1) (Malta et al., 2018). Transcriptome and clinical data from the GSE104580 cohort were used to evaluate the value of the signature to predict the transarterial chemoembolization (TACE) response. By utilization of the R package “IMvigor210CoreBiologies”, the association between the signature and the response of atezolizumab (an anti-PD-L1 blockade) was evaluated in the IMvigor210 cohort (an immunotherapy study for bladder cancer). Immunohistochemical (IHC) staining images were downloaded from The Human Protein Atlas database (HPA, https://www.proteinatlas.org/).

### DEG analysis and candidate genes selection

After classifying TCGA HCC patients into two groups based on the median mRNAsi score, the differences in OS and DFS were analyzed by using the “survminer” R package (v.4.0.3). We compared the gene expression feature of the two groups and selected genes with a false discovery rate (FDR) < 0.05 and |log_2_ fold change (FC)| > 1 as the DEG. Next, we performed a gene set enrichment analysis (GSEA) on the DEGs using GSEA software (version 4.0.1, http://www.broad.mit.edu/gsea/) and selected key pathways, and genes contained in key pathways were selected as candidate ones.

### Construction and validation of an ECM-related prognostic model

First, we used the LASSO analysis to screen variables from high dimensional data to construct an ECM signature (Gui and Li, 2005), and then we calculated the coefficients of this signature using the “glmnet” R package. The risk score formula is as follow: risk score =Σ Coefficient (ECMs i) * Expression (ECMs i). *Coefficient of gene (i)* is the regression coefficient of gene (i) in the LASSO–Cox regression model and *Expression of gene (i)* is the expression value of gene (i) for each patient.

We divided the HCC patients into high- and low-risk groups based on the median risk score and utilized the Kaplan–Meier (K-M) analysis to compare the survival differences between different risk groups; the prognostic capability of ECMs was then measured by the area under the curve (AUC) of a time-dependent receiver operating characteristic (ROC). Last, we utilized datasets from the validation cohorts to confirm this prognostic prediction capacity.

### Correlation between the risk score and clinical characteristics

We analyzed the relationship between the risk score and the clinical features of HCC including AFP, vascular invasion, primary tumor (T) stage, regional lymph nodes (N) stage, and distant metastasis (M) stage. To evaluate the stability of the robustness and accuracy of prognostic models, we compared the survival difference between high- and low-risk groups under clinical characteristic stratification.

### Construction and assessment of a predictive nomogram

We explored the correlation and independence of ECMs signature and clinical characteristics using a multivariate Cox risk regression analysis. In order to be applicable to the clinic, we integrated the risk score with vascular invasion, AFP, T stage, and N stage to construct a nomogram using the “rms” R package. The prognostic predictive ability of nomogram was evaluated by the calibration curve and time-dependent ROC curves. The ROC curves were plotted by using the “timeROC” R package. The survival net benefits of each variable were estimated by a decision curve analysis (DCA) using the “stdca” R package.

### Functional and pathway enrichment analysis

To explore biological processes related to the risk score, Reactome pathway (https://reactome.org/) and Kyoto Encyclopedia of Genes and Genomes (KEGG) pathway (https://www.genome.jp/kegg/) analyses were performed by using the “clusterProfiler” R package.

### Determination of tumor-infiltrating immune cells

CIBERSORT is a tool which can estimate the distribution of 22 common tumor-infiltrating immune cells, including B cells, T cells, NK cells, and macrophage cells, by transcriptome profiles from the TCGA cohort (Newman et al., 2015). Meanwhile, the tumor immune dysfunction and exclusion (TIDE) score was calculated using an online tool (http://tide.dfci.harvard.edu/) (Jiang et al., 2018). Patients’ epithelial–mesenchymal transition (EMT) scores were calculated according to the methods described in a previously published study (Qiu et al., 2022).

### Genomic profile analysis

Genetic mutation data were analyzed and visualized by using the ‘‘maftools’’ R package. We compared the differences of somatic variants in most prevalent mutated genes between high- and low-risk groups. The copy number variant (CNV) data were downloaded from the Affymetrix Genome-Wide Human SNP Array 6.0 platform using the “TCGAbiolinks” package, and abnormal chromosomal regions were detected using the “R/Bioconductor GAIA” package (Morganella et al., 2011). Non-synonymous mutation data were obtained from the Ensembl Variation (https://asia.ensembl.org/info/genome/variation/prediction/predicted_data.html) database. Subsequently, the data were read and the TMB values were calculated using the ‘‘maftools’’ R package.

### Prediction of treatment response

In total, four classic drugs were selected from the Genomics of Drug Sensitivity in Cancer (GDSC) database (https://www.cancerrxgene.org/) for analysis. The half-maximal inhibitory concentration (IC_50_) is an important indicator for evaluating drug efficacy or sample response to treatment. The GDSC database helps in predicting each sample’s response to targeted therapy or chemotherapy based on the sample’s transcriptome. According to the cellular expression profiles in the GDSC database, a ridge regression model was constructed with the “pRRopheticl” R package to calculate the IC_50_ of the drugs in the two risk groups. Transcriptome data and treatment response information from the GSE104580 cohort were used to test the effectiveness of signature in discriminating TACE treatment responders from non-responders. Transcriptome data and treatment response information from the IMvigor210 cohort were used to examine the effectiveness of the signature in identifying complete/partial responses (CR/PR) or stable/progressive disease (SD/PD) with immunotherapy.

### Statistical analysis

All statistical analyses in this study were based on R software (v.4.0.3). We used Fisher’s test to compare the categorical variables and the K-M curve to evaluate the differences in survival between different risk groups. The results of the multivariate Cox regression analysis were visualized using the nomogram. The concordance index, time-dependent ROC, and calibration were also important indicators used to assess the nomogram. All tests were two-sided, and *p* < 0.05 was considered statistically significant, unless otherwise stated.

## Results

### Stemness in HCC

**FIGURE 1:**
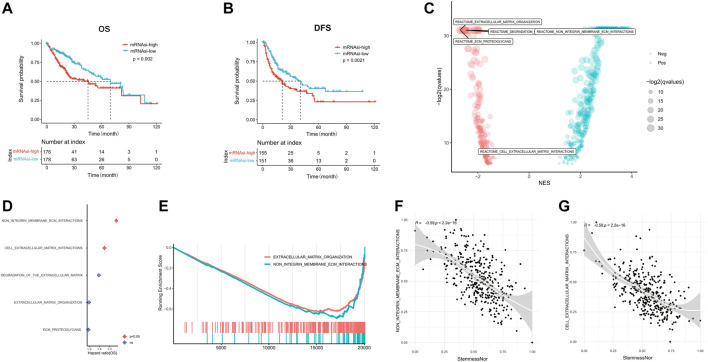
linical characteristics of stemness in HCC. The Kaplan–Meier plots illustrate the **(A)** overall survival and **(B)** disease-free survival in the high mRNAsi group compared with the low mRNAsi group. **(C)** Significantly enriched pathways of differentially expressed genes between high and low mRNAsi groups. The locations of ECM-related pathways are indicated. **(D)** Correlation analysis of ECM-related pathways and prognosis of HCC. **(E)** GSEA analysis of differentially expressed genes between high and low mRNAsi groups. **(F)** Correlation of the non-integrin membrane–ECM interaction pathway with the mRNAsi score. **(G)** Correlation of the cell–extracellular matrix interaction pathway with the mRNAsi score. HCC, hepatocellular carcinoma; mRNAsi, mRNA expression–based stemness index; ECM, extracellular matrix; GSEA, Gene Set Enrichment Analysis.

According to the median mRNAsi score, we dichotomized HCC patients in the training set into mRNAsi-high and mRNAsi-low groups and identified that HCC patients with high mRNAsi had both significantly shorter OS (median OS: 45.11 vs. 69.57 months, *p* = 0.002; Figure 1A) and DFS (median DFS: 20.91 vs. 40.41 months, *p* = 0.0021; Figure 1B). The functional enrichment analysis of the DEGs between mRNAsi-high and mRNAsi-low groups revealed that ECM-related pathways were the most significantly enriched (Figure 1C). Among those pathways, non-integrin membrane–ECM interactions and cell–ECM interactions pathways were regarded as the key pathways (*p* < 0.05, Figures 1D,E) with 75 ECM-related genes kept in the two pathways (Supplementary Table S2). The expression levels of these 75 ECM-related genes were mainly negatively correlated with tumor cell stemness (*p* < 0.05, Supplementary Figure S2A). In addition, we also found that non-integrin membrane–ECM interactions and cell–ECM interactions pathways were significantly negatively related with the stemness index (R = −0.59 and −0.58, respectively, *p* < 0.001; Figures 1F,G).

### Construction of the ECM-related prognostic model

**FIGURE 2:**
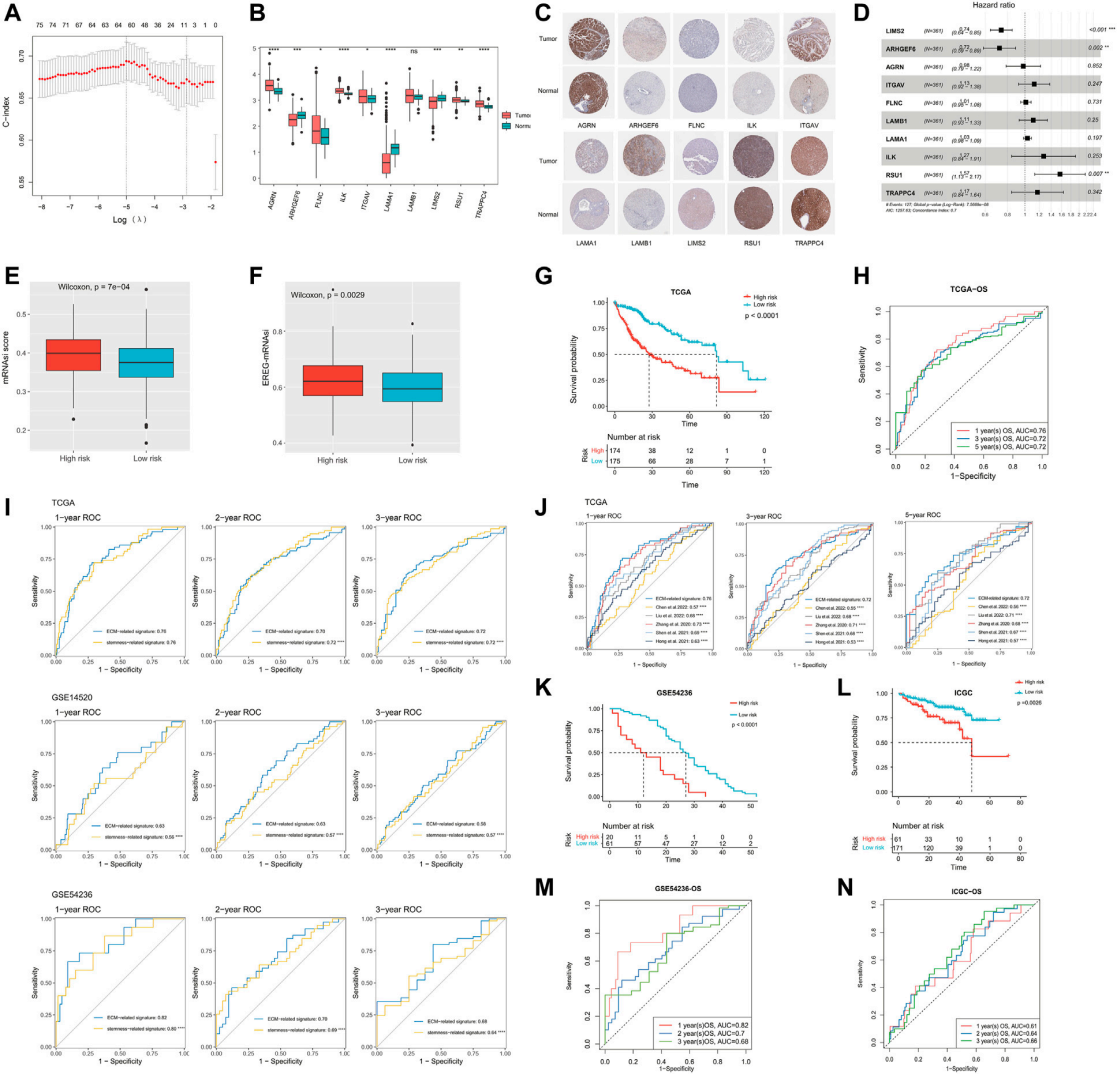
Construction and validation of the ECM-related prognostic model. **(A)** LASSO regression analysis showed the partial likelihood deviation curve of the minimum number genes corresponding to the covariates (the abscissa represents the CI of each lambda, and the ordinate represents errors in cross-validation). **(B)** Expression analysis of the 10 genes (*TRAPPC4*, *RSU1*, *ILK*, *LAMA1*, *LAMB1*, *FLNC*, *ITGAV*, *AGRN*, *ARHGEF6*, and *LIMS2*) in HCC samples and normal samples. **(C)** Representative immunohistochemical staining plots for 10 selected genes. **(D)** Univariate Cox regression analysis of these 10 genes. **(E)** Comparison of mRNAsi scores between high- and low-risk groups. **(F)** Comparison of EREG-mRNAsi scores between high- and low-risk groups. **(G)** Kaplan–Meier curves showing different overall survival of patients in high- and low-risk groups. **(H)** ROC curves of ECM-related signature for predicting the 1/3/5-year overall survival in TCGA. **(I)** Performance comparison of ECM-related signature and stemness-related signature (constructed based on the DEGs between the mRNAsi-high and mRNAsi-low groups). **(J)** Performance comparison of ECM-related signature and published stemness-related signatures. **(K)** Kaplan–Meier curves showing different overall survival of patients in high- and low-risk groups based on GSE54236. **(L)** Kaplan–Meier curves showing different overall survival of patients in high- and low-risk groups based on ICGC. **(M)** ROC curves of ECM-related signature for predicting the 1/2/3-year overall survival in GSE54236. **(N)** ROC curves of ECM-related signature for predicting the 1/2/3-year overall survival in ICGC. LASSO, least absolute shrinkage and selection operator; mRNAsi, mRNA expression–based stemness index; ROC, receiver operating characteristic; ECM, extracellular matrix; TCGA, The Cancer Genome Atlas; IGGC, International Cancer Genome Consortium; DEG, Differentially Expressed Gene.

Subsequently, we performed the LASSO regression analysis on the 75 ECM-related genes screened before and finally selected 10 genes (*LIMS2*, *ARHGEF6*, *AGRN*, *ITGAV*, *FLNC*, *LAMB1*, *LAMA1*, *ILK*, *RSU1*, and *TRAPPC4*) to construct a prognostic prediction model (Figure 2A). The 10 genes were found to be expressed in both tumor and normal tissues, among which *AGRN*, *FLNC*, *ILK*, *ITGAV*, *RSU1*, and *TRAPPC4* were significantly upregulated in the tumor tissues, while *ARHGEF6*, *LAMA1*, and *LIMS2* were significantly downregulated in the tumor tissues (*p* < 0.05, Figure 2B). We downloaded representative images of IHC staining of the proteins encoded by these genes in the TCGA-LIHC cohort from the HPA database. The data showed that the expression levels of these proteins in tumor tissues were higher than those in normal tissues, except for the *LIMS2* protein, which was consistent with mRNA expression (Figure 2C). Matrix metalloproteinase (MMP) is a family of zinc-dependent ECM remodeling endopeptidases with the ability to degrade almost all components of the ECM (Cabral-Pacheco et al., 2020). We found that 10 hub ECM-related genes in this study were significantly correlated with most members of the MMP family at the mRNA expression level (*p* < 0.05, Supplementary Figure S2B). Furthermore, the forest plots showed that the overexpression of *LIMS2* (HR = 0.74, 95% CI = 0.64–0.85, and *p* < 0.001) and *ARHGEF6* (HR = 0.72, 95% CI = 0.59–0.89, and *p* = 0.002) were related to a significantly superior OS; on the contrary, the upregulation of *RSU1* was significantly associated with a worse survival (HR = 1.57, 95% CI = 1.13–2.17, and *p* = 0.007; Figure 2D). The risk scores were calculated for each patient based on the gene expression levels and Cox regression coefficients.

Risk score= (−0.227 * Exp_*LIMS2*_) + (−0.008 * Exp_*ARHGEF6*_) + (0.002 * Exp_*AGRN*_) + (0.004 * Exp_*ITGAV*_) + (0.005 * Exp_*FLNC*_) + (0.007 * Exp_*LAMB1*_) + (0.015 * Exp_*LAMA1*_) + (0.043 * Exp_*ILK*_) + (0.131 * Exp_*RSU1*_) + (0.132 * Exp_*TRAPPC4*_).

As the risk score increased, the stemness of HCC got stronger. The median mRNAsi score in the high-risk group was significantly higher than that in the low-risk group (*p* < 0.0001, Figure 2E). In addition, EREG-mRNAsi is also an index to assess tumor stemness (Malta et al., 2018). Likewise, the median EREG-mRNAsi in the high-risk group was significantly higher than that in the low-risk group (*p* = 0.0029, Figure 2F). Meanwhile, HCC patients in the high-risk group had a significantly worse prognosis than that in the low-risk group (median OS: 27.52 vs. 81.73 months, and *p* < 0.0001; Figure 2G). The AUCs of 1-, 3-, and 5-year OS in the TCGA cohort were 0.76, 0.72, and 0.72, respectively (Figure 2H). In addition, we constructed a stemness-related prognostic signature based on the DEGs between the mRNAsi-high and mRNAsi-low groups (Supplementary Figures S3A–C). The results showed that the ECM-related signature was more accurate and robust than the stemness-related signature (*p* < 0.0001, Figure 2I). Also, the performance of the ECM-related signature was also better than other published stemness-related signatures (*p* < 0.0001, Figure 2J) (Zhang et al., 2020; Hong et al., 2021; Shen et al., 2021; Chen et al., 2022; Liu et al., 2022). We further explored the correlation between our prognostic model and ECM collagen or non-collagen genes. The association of 10 ECM-related genes with collagen or non-collagen was consistent. The expressions of *LAMA1*, *LAMB1*, *ARHGEF6*, *ITGAV*, *FLNC*, and *RSU1* were significantly positively correlated with most collagen and non-collagen genes, while the expressions of *LIMS2*, *TRAPPC4*, and *ILK* were significantly negatively correlated with most collagen and non-collagen genes (*p* < 0.05, Supplementary Figure S2C).

### Validation of the ECM-related prognostic prediction model

To validate the accuracy of the ECM-related prognostic prediction model, we performed a confirmatory test using three independent validation sets from the GEO and ICGC databases. The K-M curve showed that there was a significant difference in OS between the high- and low-risk groups, and the prognosis of patients in the high-risk group was significantly worse than that in the low-risk group, consistent with the results of the training cohort (GSE54236, median OS 12 vs. 27 months, and *p* < 0.0001; ICGC-LIRI-JP, median OS 48 vs. not reached, and *p* = 0.0026; Figures 2K,l). The ROC curves showed that the AUC of the 1-, 2-, and 3-year survival time of the GSE54236 cohort was 0.82, 0.7, and 0.68, respectively; the AUC of the 1-, 2-, and 3-year survival time of the ICGC cohort was 0.61, 0.64, and 0.66, respectively (Figures 2M,N). Overall, the ECM-related prognostic model could stably and accurately predict the prognosis of HCC patients. Furthermore, the tumor volume doubling time was significantly negatively correlated with the risk score in HCC patients in the GSE54236 cohort (*p* = 0.00076, Supplementary Figure S4).

### Association with the clinical characteristics

**FIGURE 3:**
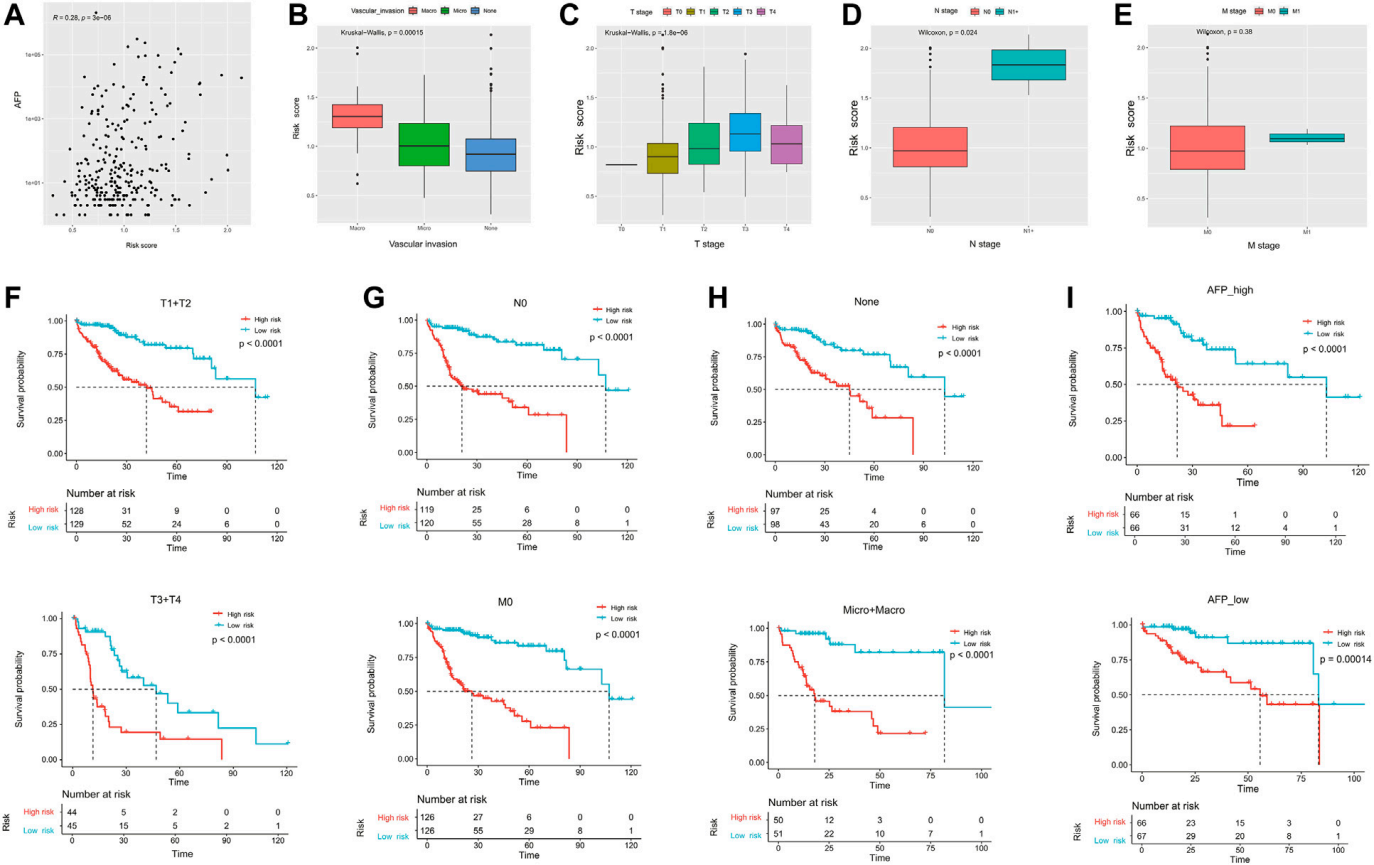
Correlation of ECM-related prognostic models with clinicopathological parameters. The correlation of the risk score and **(A)** AFP, **(B)** vascular invasion, **(C)** primary tumor stage, **(D)** regional lymph nodes stage, and **(E)** distant metastasis stage. **(F–I)** Among the different stratified subgroups, the high-risk group had a poor prognosis. AFP, *α*-fetoprotein.

As shown in Figure 3A, the risk score was positively correlated with the AFP level (R = 0.28, p = 3e^−6^). In addition, the risk score was significantly higher in the HCC patients presented with macro-vascular invasion, followed by those with micro-vascular invasion (Figure 3B). Meanwhile, HCC patients with the advanced tumor stage (T) and the positive lymph node stage (N) had significantly higher risk scores (Figures 3C,D). However, as there were limited number of patients with long distant metastasis, there was no significant difference between patients presented with the long distant metastasis (M) stage or not (*p* = 0.38, Figure 3E).

We subsequently divided the clinical features into different subgroups to observe the stability of the ECM prediction efficacy. The results showed that regardless of whether in the advanced tumor stage (T3+T4) or not (T1+T2), patients in the high-risk group had significantly worse OS (Figure 3F). Meanwhile, the trend was similar in patients with N0 (median OS: 21.0 vs. 107.1 months and *p* < 0.0001) and/or M0 (median OS: 26.4 vs. 107.1 months and *p* < 0.0001; Figure 3G). Last, the ECM signature could successfully differentiate HCC patients with worse outcomes regardless of whether present with vascular invasion or not, or whether with high or low AFP levels (Figures 3H,I).

### Establishment of a nomogram integration with independent predictive factors

**FIGURE 4:**
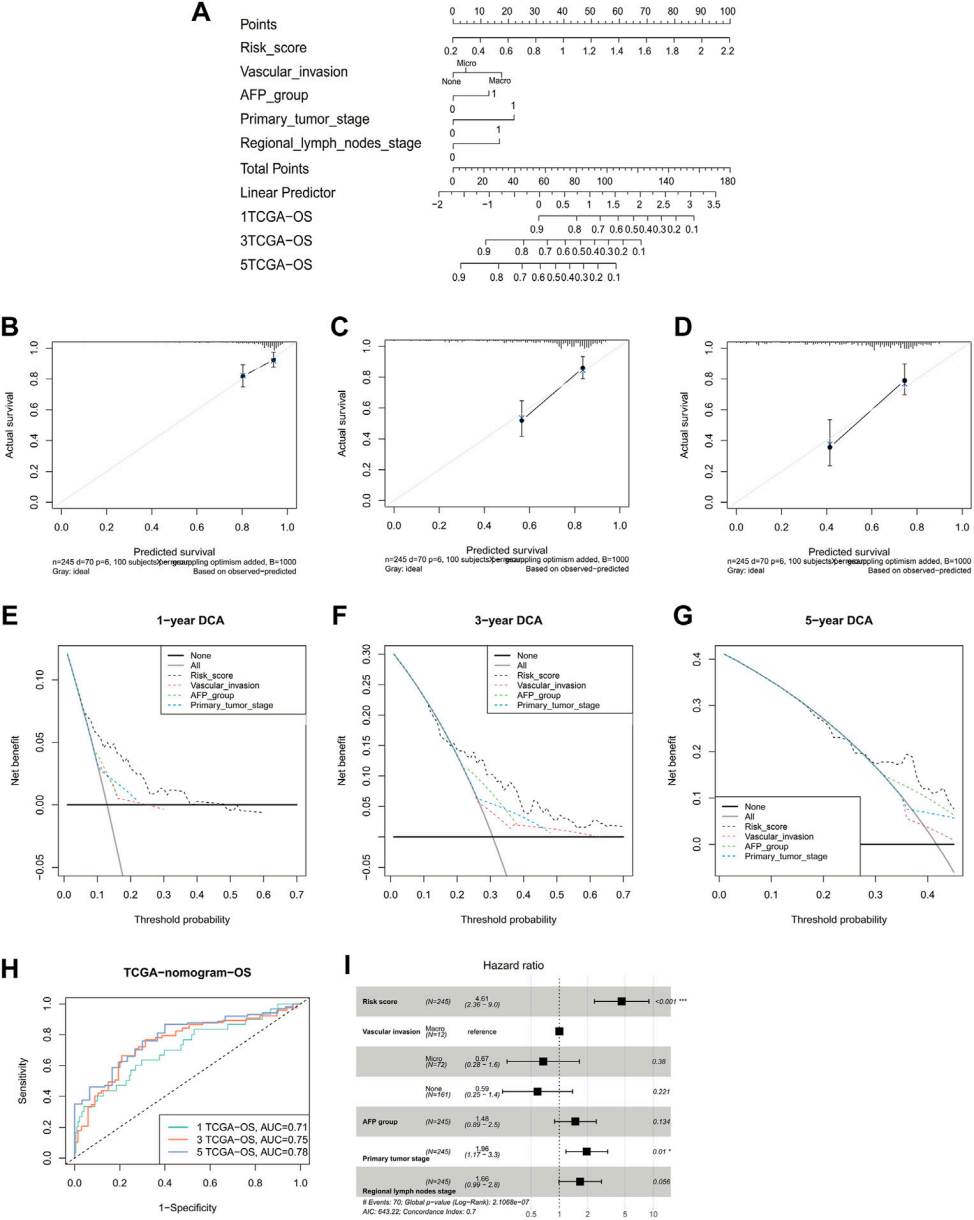
Validation of a nomogram-integrated independent predictive factors. **(A)** Nomogram with a combination of risk scores and different clinical features. A calibration plot for predicting the accuracy of the nomogram in **(B)** 1-, **(C)** 3-, **(D)** and 5-year. **(E–G)** 1-, 3-, and 5-year DCA analysis of the nomogram. **(H)** ROC curves of the nomogram for predicting the 1/3/5-year overall survival in TCGA. **(I)** Multivariate Cox regression analyses of the risk score and clinicopathological factors for overall survival in TCGA. DCA, decision curve analysis; ROC, receiver operating characteristic; TCGA, The Cancer Genome Atlas.

To better predict the 1-, 3-, and 5-year survival of HCC patients, we constructed a nomogram combining the risk score, vascular invasion, AFP, T stage, and N stage. The C-index of nomogram was 0.70 (95% CI: 0.63–0.77) (Figure 4A). Meanwhile, it could be observed that the calibration curves at 1-, 3-, and 5-year survival showed a good consistency between actual observation and the prediction by nomogram (Figures 4B–D). The results showed the net benefit of nomogram-assisted decisions at a wide range of threshold probabilities, suggesting potential clinical usefulness of the nomograms (Figures 4E–G). The AUCs for the 1-, 3-, and 5-year survival of the constructed nomogram were 0.71, 0.75, and 0.78, respectively (Figure 4H). Finally, the multivariate Cox regression analysis indicated that the risk score and the T stage were the only two independent risk factors (HR = 4.61, 95% CI = 2.36–9.0, and *p* < 0.001; HR = 1.96, 95% CI = 1.17–3.3, and *p* = 0.01; Figure 4I).

### Biological pathways and functional enrichment analysis

**FIGURE 5:**
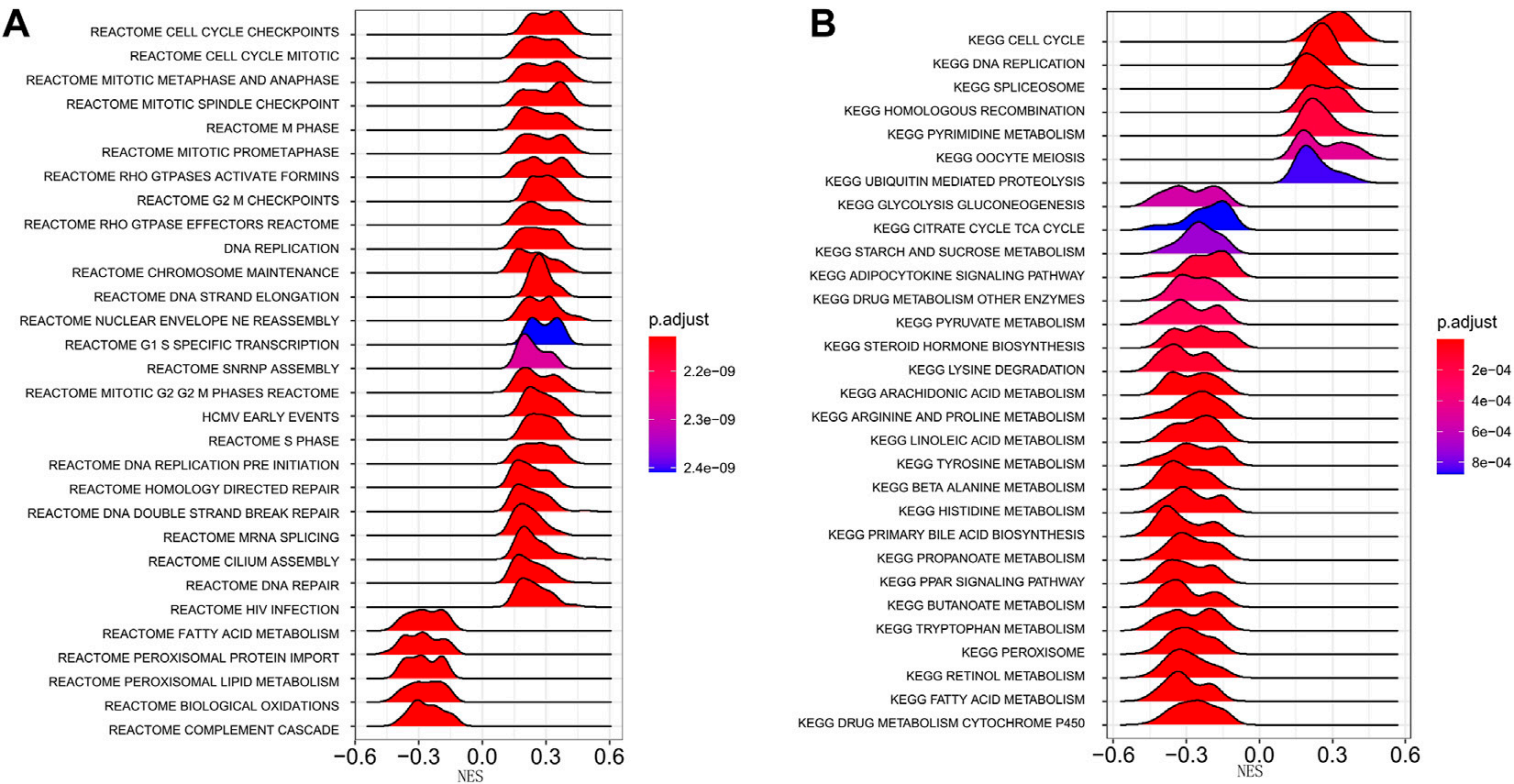
Enrichment analysis of differentially expressed genes between high- and low-risk groups. **(A)** Reactome pathway enrichment analysis. **(B)** KEGG pathway enrichment analysis. The peak area and color represent the absolute value of NES and the q-values, respectively. KEGG, Kyoto Encyclopedia of Genes and Genomes; NES, Normalized Enrichment score.

We performed a pathway enrichment analysis using DEGs between high- and low-risk groups in the TCGA-LIHC cohort to explore the biological processes associated with the risk scores. A Reactome pathway enrichment results showed that DEGs were significantly enriched in the cell cycle and metabolism-related pathways, such as cell cycle checkpoints, cell cycle mitosis, DNA replication, fatty acid metabolism, and peroxisome lipid metabolism pathways (*p* < 0.05, Figure 5A). The KEGG pathway enrichment results were consistent with the Reactome enrichment, which were also dominated by the cell cycle and metabolism-related pathways, including cell cycle, DNA replication, homologous recombination, pyruvate metabolism, tryptophan metabolism, and starch and sucrose metabolism pathways (*p* < 0.05, Figure 5B).

### Immune landscape, immune checkpoint profile, and immunotherapy response prediction

**FIGURE 6:**
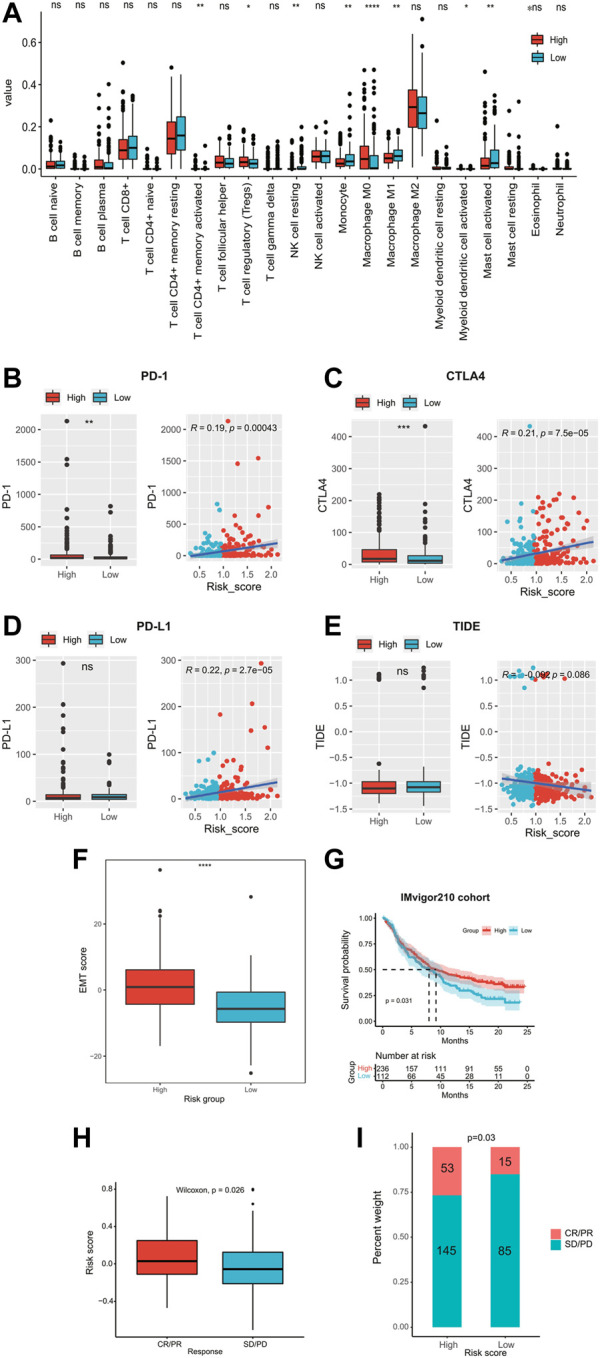
Immune landscape, immune checkpoint profile, and immunotherapy response prediction. **(A)** Comparison of the proportion of 22 tumor-infiltrating immune cells in the high- and low-risk groups. The expression levels of immune checkpoints **(B)** PD-I, **(C)** CTLA4, and **(D)** PD-L1 in high- and low-risk groups. **(E)** Comparison of TIDE scores in high- and low-risk groups. **(F)** Comparison of EMT scores in high- and low-risk groups. **(G)** Kaplan–Meier curves of patients receiving immunotherapy in the IMvigor210 cohort. **(H–I)** Relationship of the risk score and immunotherapy sensitivity. TIDE, tumor immune dysfunction and exclusion; EMT, epithelial–mesenchymal transition; ECM, extracellular matrix; AUC, area under the curve.

We evaluated the tumor-infiltrated immune cell contents and immune checkpoint profile and predicted immunotherapy responses in different risk groups. In total, the high-risk group had a significantly higher abundance of macrophage M0, resting mast cells, and Treg cells and lower abundance of activated CD4^+^ memory T cells, resting NK cells, monocytes, macrophage M1, activated myeloid dendritic cells, and activated mast cells (Figure 6A). Common immune checkpoints such as PD-1 (Figure 6B) and CTLA-4 (Figure 6C) were significantly upregulated in the high-risk groups and their expression levels were significantly positively correlated with the risk scores. However, the expression level of PD-L1 was not significantly different between the high- and low-risk groups (Figure 6D). In addition, there was no significant difference in the TIDE score between high- and low-risk groups (Figure 6E). In addition, the high-risk group had significantly higher EMT scores than those of the low-risk group (*p* < 0.0001, Figure 6F). We validated the prediction value of the ECMs signature for immunotherapy response of the patients in the IMvigor210 cohort. The K-M survival curve showed that immunotherapy-treated patients in the high-risk group had significantly longer OS (median OS 9.2 vs. 8.0 months, *p* = 0.031; Figure 6G). Moreover, the risk score of immunotherapy patients with objective response (CR/PR status) was significantly higher than those without it (SD/PD, *p* = 0.026; Figure 6H). Also, the proportion of immunotherapy patients with objective response was significantly higher in the high-risk group (*p* = 0.03, Figure 6I).

### Genomic profile related to the ECM prognostic signature

**FIGURE 7:**
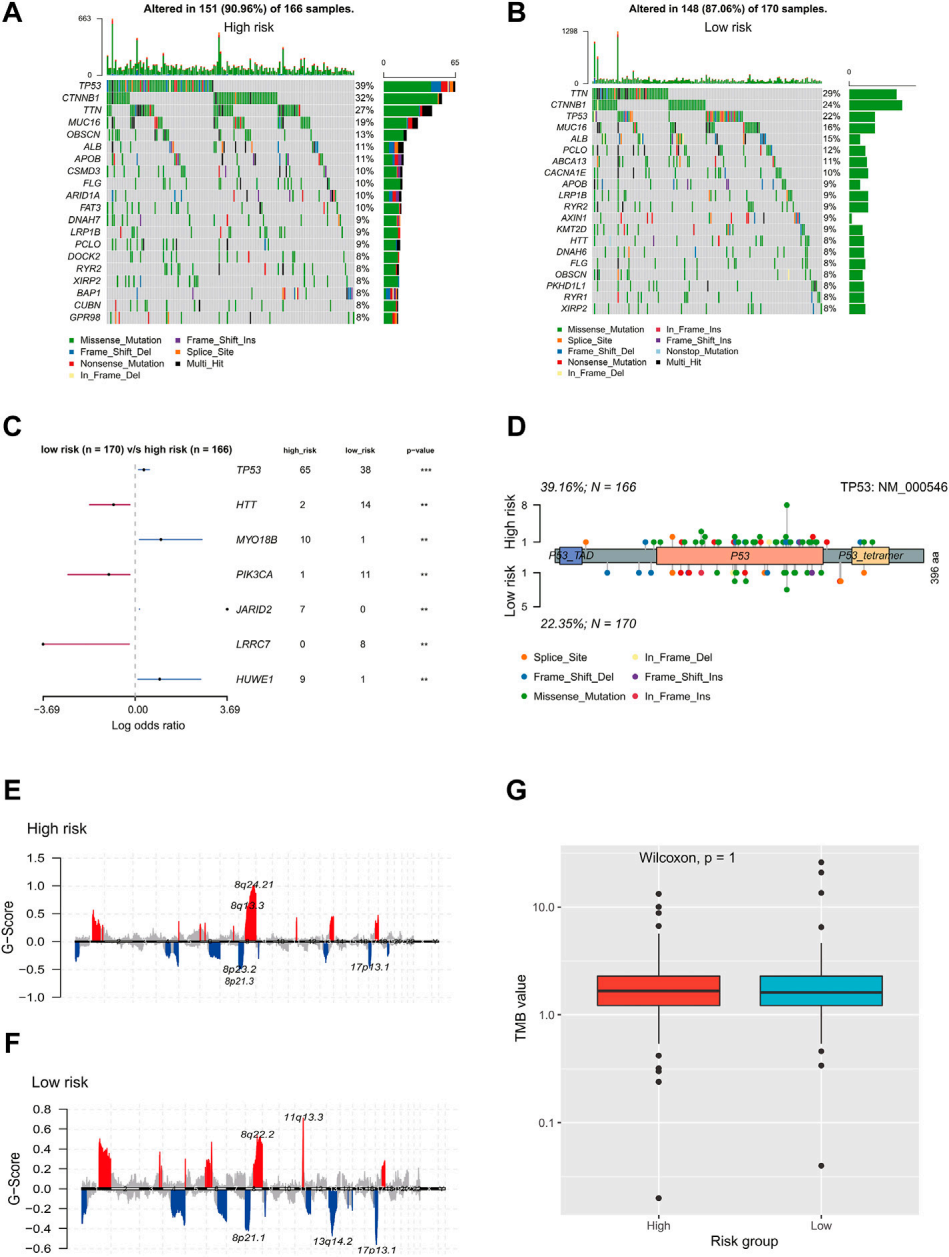
Genomic landscape of HCC patients in the high- and low-risk group. Oncoprints showed the most prevalent alter genes in **(A)** high-risk and **(B)** low-risk. **(C)** Significantly different mutated genes between low- and high-risk groups. **(D)** Distribution of TP53 alterations in low- and high-risk groups. **(E,F)** Detection and comparison of significant amplifications and deletions of copy number between high- and low-risk groups. **(G)** Difference in the TMB values between low- and high-risk groups. HCC, hepatocellular carcinoma; TMB: tumor mutation burden.

There are two figures presenting the top 20 frequently mutated genes in the high-risk and low-risk groups (Figures 7A,B). The prevalence of *TP53*, *MYO18B*, *JARID2*, and *HUWE1* alterations in the high-risk group was significantly higher than those in the low-risk group; on the contrary, *HTT*, *PIK3CA*, and *LRRC7* mutations were more enriched in the low-risk group (*p* < 0.01, Figure 7C). As shown in Figure 7D, *TP53* alterations were mainly located in the P53 domain, and the high-risk group had more mutations than the low-risk group in this domain. In addition, the low-risk group had significantly high frequencies of amplifications and deletions in chromosome 11 and 13, respectively (FDR<0.01, Figures 7E,F). There was no significant difference in the TMB value between the two risk groups (*p* = 1, Figure 7G).

### Chemotherapy response prediction

**FIGURE 8:**
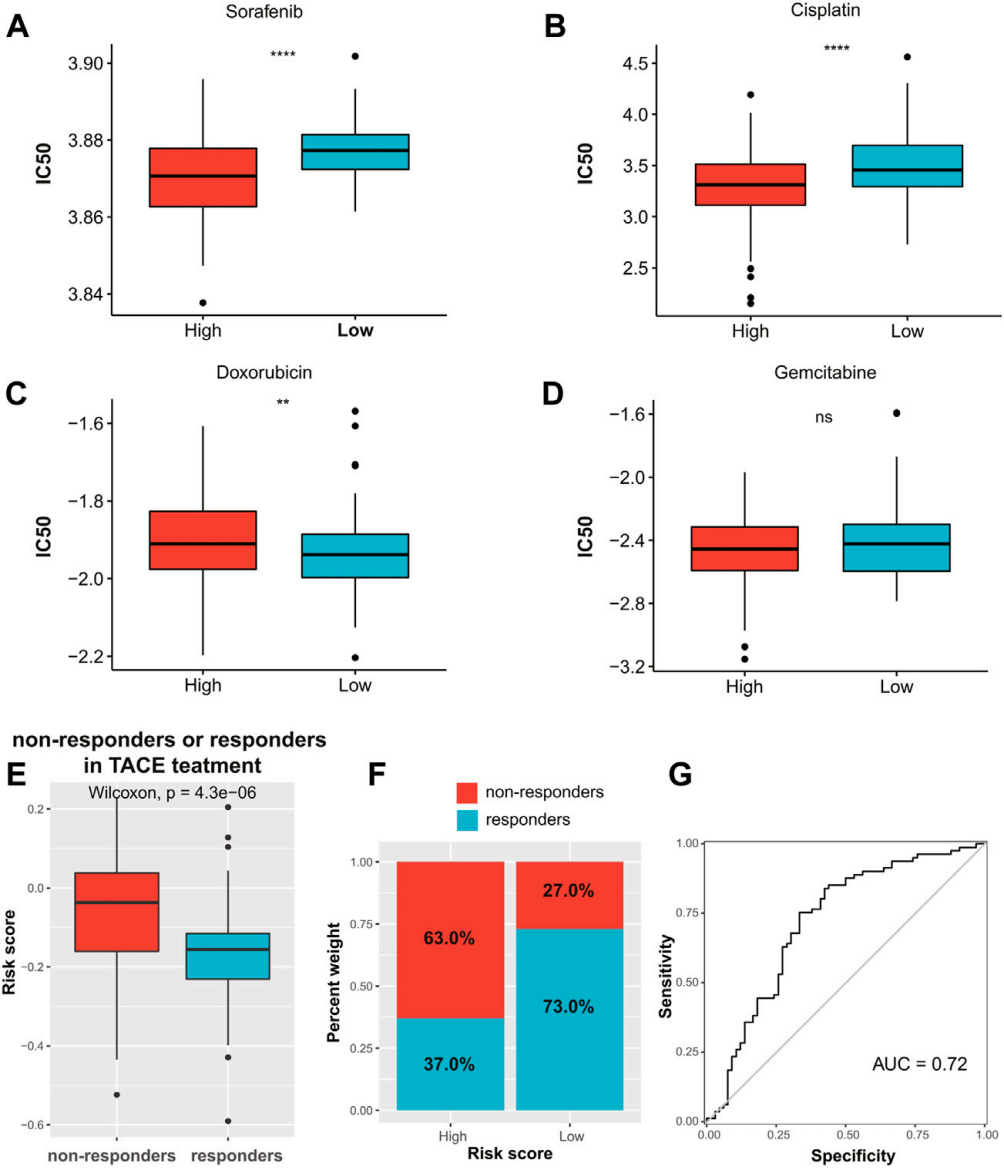
Chemotherapy response prediction. The boxplots of the estimated IC_50_ for **(A)** sorafenib, **(B)** cisplatin, **(C)** doxorubicin, and **(D)** gemcitabine in the high- and low-risk groups. **(E)** Distribution of risk scores among TACE treatment responders and non-responders in the GSE104580 cohort. **(F)** Proportion of TACE treatment responders or non-responders in the high- and low-risk groups in the GSE104580 cohort. **(G)** ROC curve of ECM-related signature in predicting TACE response in the GSE104580 cohort. IC_50_, half-maximal inhibitory concentration; TACE, transarterial chemoembolization; ROC, receiver operating characteristic; ECM, extracellular matrix.

The results of drug sensitivity analysis indicated that the patients in the high-risk group were more sensitive to commonly used drugs such as sorafenib and cisplatin (*p* < 0.0001, Figures 8A,B), but more resistant to doxorubicin (*p* < 0.01, Figure 8C). Meanwhile, there was no significant difference in sensitivity to gemcitabine between the two risk groups (Figure 8D). In addition, we assessed whether the ECM signature could predict the TACE benefit for HCC patients. The boxplots showed that non-responders of TACE had significantly higher risk scores than responders (Figure 8E). We also assessed the distribution of non-responders and responders in different risk groups (Figure 8F) and found that patients in the low-risk group had higher sensitivity to TACE than those in the high-risk group. The AUC of the ECMs signature to predict response to TACE in HCC patients was 0.72 (Figure 8G).

## Discussion

The resistance of HCC to traditional treatments and high tumor recurrence after therapy are pivotal causes of cancer-related deaths (Liu et al., 2020); among this process, CSCs play key roles through genetic mutations, dysregulation of signaling pathways, epigenetic disruption, and/or TME regulation (Liu et al., 2020). In this study, by analyzing the characteristics of CSCs in HCC, we found that ECM is closely related to the occurrence and development of HCC. The ECM is the major structural component of the TME and is a highly dynamic structure (Nallanthighal et al., 2019). ECM is both a structural scaffold and a regulator of cell signal transduction in tissues (Dalton and Lemmon, 2021). Research in recent years has mainly focused on uncovering the cellular signal transduction mechanisms involved in the development of HCC. Gene modification plays an important role in tumorigenesis, and anomalies of many related signal transduction pathways, such as MAPK pathway, PI3K/AKT/mTOR pathway, WNT/β-catenin pathway, and JAK/STAT pathway, are also related to the progression of HCC (Mir et al., 2021). A previous study has supported that ECM-related proteins establishes a physical and biochemical niche for CSCs, providing structural and biochemical support for regulating CSCs proliferation, self-renewal, and differentiation (Nallanthighal et al., 2019). A total of 10 key ECM-related genes were obtained from ECM-related pathways to construct a novel prognostic prediction signature, including *TRAPPC4*, *RSU1*, *ILK*, *LAMA1*, *LAMB1*, *FLNC*, *ITGAV*, *AGRN*, *ARHGEF6*, and *LIMS2*. Previous studies have demonstrated that these 10 ECM-related genes are associated with the prognosis of various types of cancer, especially HCC. In published studies, *AGRN* (Zou et al., 2019), *ITGAV* (Zhang et al., 2019; Weiler et al., 2020), *FLNC* (Qi et al., 2016; Yang et al., 2017), *ILK* (Chan et al., 2011), and *RSU1* (Gkretsi and Bogdanos, 2015) were overexpressed in HCC tumor tissues, thereby promoting tumor development, metastasis, and even leading to poor prognosis, which is consistent with our findings. These genes were more upregulated in the high-risk group, which was responsible for causing the inferior outcomes of patients with the high-risk scores. When these genes were knocked out or downregulated, the proliferation and metastasis of HCC cells could be successfully inhibited, which indicates that they also have the potential to be developed as new therapeutic targets (Yang et al., 2017; Zou et al., 2019). Contrary to our findings, studies have shown that *TRAPPC4* is expressed at low levels in HCC tissues, and HCC patients with low *TRAPPC4* expression have a shorter survival time than those with high expression (Shen et al., 2018). This difference may be due to the tumor heterogeneity of HCC. In addition, *LIMS2* and *ARHGEF6* were expressed at lower levels in tumors than in normal tissues in our study and associated with better clinical outcomes. The dysregulation of *LIMS2*, also known as *PINCH2*, caused liver enlargement and tumorigenesis (Donthamsetty et al., 2013).

Subsequently, we explored the probability of the ECM signature in clinical application. By evaluating the relationship between clinical features and the risk score, we found that ECM signature was closely associated with AFP, vascular invasion, T stage, and N stage. AFP is the most prevalent clinically applied biomarker for the detection and treatment monitor of HCC, associated with HCC differentiation (Tangkijvanich et al., 2000). Previous studies found that the components of ECM, such as fibroblasts, would stimulate the paracrine and upregulate the AFP expression level (Ogunwobi et al., 2019). Meanwhile, vascular invasion has been reported not only to be present in about 50% of HCC but also to be a major risk factor for disease recurrence, associated with shorter survival in HCC, which is consistent with our results (Krishnan et al., 2021). The positive correlation between ECM and vascular invasion may be mainly attributed to the reason that ECM provides an essential scaffold supporting the vascular endothelium (Davis and Senger, 2005). Meanwhile, ECM induces tumor cell to transfer to an endothelial-like phenotype, imitating the vasculature that connect to blood vessels; and on the other hand, hypoxic tumor microenvironment also facilitates the ECM to release VEGFR and further angiogenic events (Winkler et al., 2020).

In recent years, immune-based therapies have revolutionized the systematic management of advanced cancers (Whiteside et al., 2016). The application of immune checkpoint inhibitor (ICI) therapy targeting PD-1, PD-L1, or CTLA-4 represents a major breakthrough for many types of cancers, including HCC (Hoos, 2016). However, the objective response rate of these agents as monotherapy for HCC is only 15%–20% (Cheng et al., 2020). Previously identified biomarkers such as PD-L1 expression and tumor mutation burden cannot reliably predict the benefit of ICI therapy (Mushtaq et al., 2018). Therefore, strategies to improve their efficacy through patient stratification, or selection of potential combinations are still urgently needed. The composition of the tumor microenvironment has been shown to influence ICI response (Petitprez et al., 2020). In this study, we found that HCC patients with high-risk scores have higher sensitivity to ICIs. However, there was no significant difference in tumor mutation counts or in T cell function (revealed by the TIDE algorism) between high- and low-risk groups. Then this correlation between the risk score and ICI response may be interpreted as the positive correlation between the risk score and the expression of immune checkpoint genes, including PD-1 and CTLA4. Previous studies have shown that tumor-infiltrating lymphocytes, such as Treg cells and tumor-associated macrophages, play an important role in driving immune evasion, which in turn drives HCC progression and affects patient treatments (Lurje et al., 2021). In our study, there were significant differences in the abundance of immune cell infiltration between the two groups of patients, including CD4^+^ T cells, Treg cells, macrophages M0, and myeloid dendritic cells. It has been reported that the number of Tregs in tumor tissue or peripheral blood of HCC patients is increased compared with healthy individuals (Oura et al., 2021), and Tregs are associated with poorer median OS (Lim et al., 2019). Both macrophages and myeloid dendritic cells belong to myeloid cells, which are important components of tumor tissues and key regulators of the immune environment (Wu et al., 2020). They promote tumor development and are associated with prognosis in HCC patients (Wu et al., 2020). These findings explain the poor prognosis of the high-risk group in our study from the perspective of the immune environment. This shows the potential of our signature in predicting the HCC tumor immune microenvironment, which may be beneficial for immunotherapy of this malignancy. Different numbers, phenotypes, and localization of tumor-infiltrating lymphocytes are not only key regulators of disease progression but also potential biomarkers for predicting immunotherapy response (Maibach et al., 2020). Previous studies have shown that higher levels of immune cell infiltration are positively associated with immunotherapy response in multiple tumor types (Karn et al., 2017; Kümpers et al., 2019). Interestingly, the high-risk group not only had higher levels of immune cell infiltration in our study but also showed a better response to immunotherapy. Therefore, ECMs signature had the potency for assisting oncologists to decide which patients are likely to respond to ICI in order to take the best course of treatment.

In conclusion, this study developed a prognostic signature composed of 10 ECM-related genes that can accurately and robustly predict the prognosis of HCC. These genes have the potential to be developed as therapeutic targets for HCC. Also, this ECMs signature has an important value for HCC in the selection of treatment.

## Data Availability

The original contributions presented in the study are included in the article/Supplementary Material; further inquiries can be directed to the corresponding author.

## Supplementary material

Supplementary Figure S1 Schematic illustration of study’s design. This study is mainly divided into three parts: first, identifying ECM-related genes; second, constructing a prognostic signature based on ECM-related genes; and finally analyzing the multi-omics features underlying the signature. ECM, extracellular matrix.

Supplementary Figure S2 (A) Correlation analysis of 75 ECM-related genes with stemness. (B) Correlation analysis of 10 selected ECM-related genes and MMP family. (C) Correlation analysis of 10 selected ECM-related genes and collagen or non-collagen. ECM, extracellular matrix; MMP, matrix metalloproteinase.

Supplementary Figure S3 Performance comparison of ECM-related prognostic prediction signature and stemness-related prognostic prediction signatures. (A) LASSO regression analysis showed the partial likelihood deviation curve of the minimum number genes corresponding to the covariates. (B) Kaplan–Meier curves showing different overall survival of patients in high- and low-risk groups. (C) ROC curves of stemness-related signature for predicting the 1/3/5-year overall survival. ECM, extracellular matrix; LASSO, least absolute shrinkage and selection operator; ROC, receiver operating characteristic.

Supplementary Figure S4 Correlation analysis of the tumor doubling time and the risk score.

## Glossary

HCC: hepatocellular carcinoma
TCGA: The Cancer Genome Atlas
GEO: Gene Expression Omnibus
ICGC: International Cancer Genome Consortium
mRNAsi: mRNA expression–based stemness index
DEGs: differentially expressed genes
ECM: extracellular matrix
LASSO: least absolute shrinkage and selection operator
ROC: receiver operating characteristic
TACE: transarterial chemoembolization
LC: liver cancer
CSCs: cancer stem cells
TME: tumor microenvironment
TCGA-LIHC: The Cancer Genome Atlas Liver Hepatocellular Carcinoma
OS: overall survival
DFS: disease-free survival
OCLR: one-class logistic regression
GSEA: gene set enrichment analysis
K-M: Kaplan–Meier
AUC: area under the curve
T stage: primary tumor stage
N stage: regional lymph nodes stage
M stage: distant metastasis stage
KEGG: Kyoto Encyclopedia of Genes and Genomes
TIDE: tumor immune dysfunction and exclusion
EMT: Epithelial–mesenchymal transition
CNV: copy number variant
GDSC: Genomics of Drug Sensitivity in Cancer
CR/PR: complete/partial responses
SD/PD: stable/progressive disease
DCA: decision curve analysis.
