# Supplementary figures and images for: Stemness analysis in hepatocellular carcinoma identifies an extracellular matrix gene–related signature associated with prognosis and therapy response
Source: Front Genet. 2022 Aug 30;13:959834. doi: 10.3389/fgene.2022.959834 (PMC9468756; doi:10.3389/fgene.2022.959834)

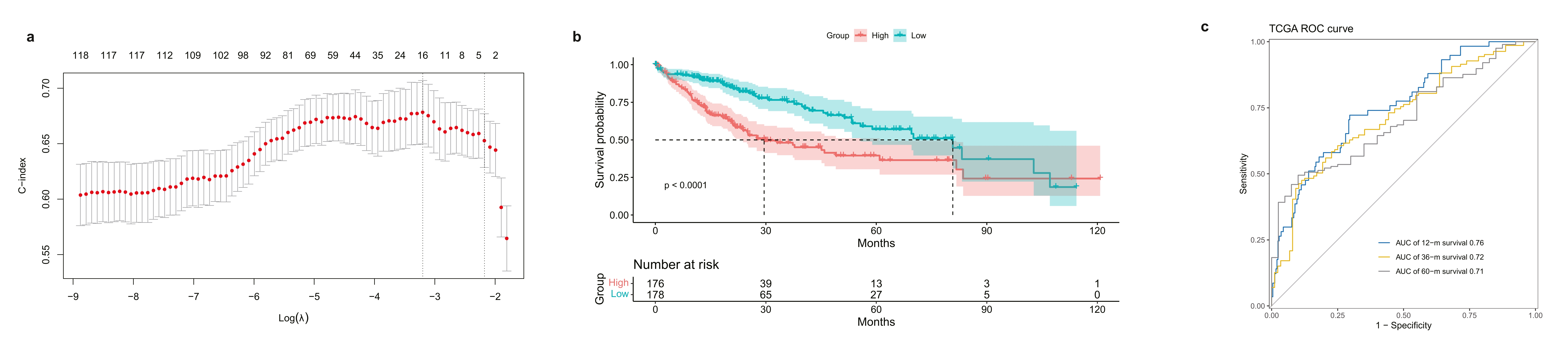

Supplement: Supplementary file 1 [file Image3.JPEG]

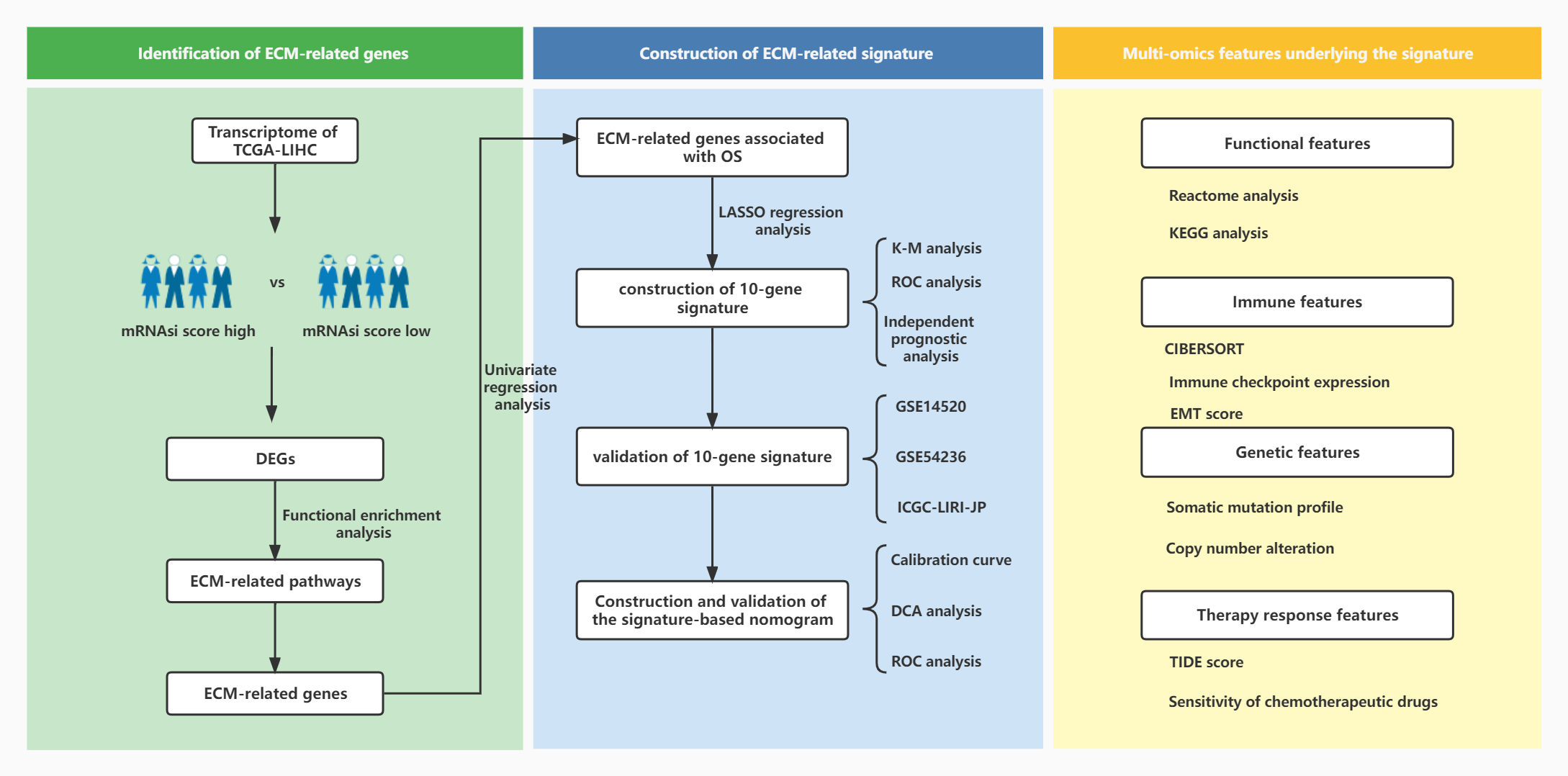

Supplement: Supplementary file 3 [file Image1.JPEG]

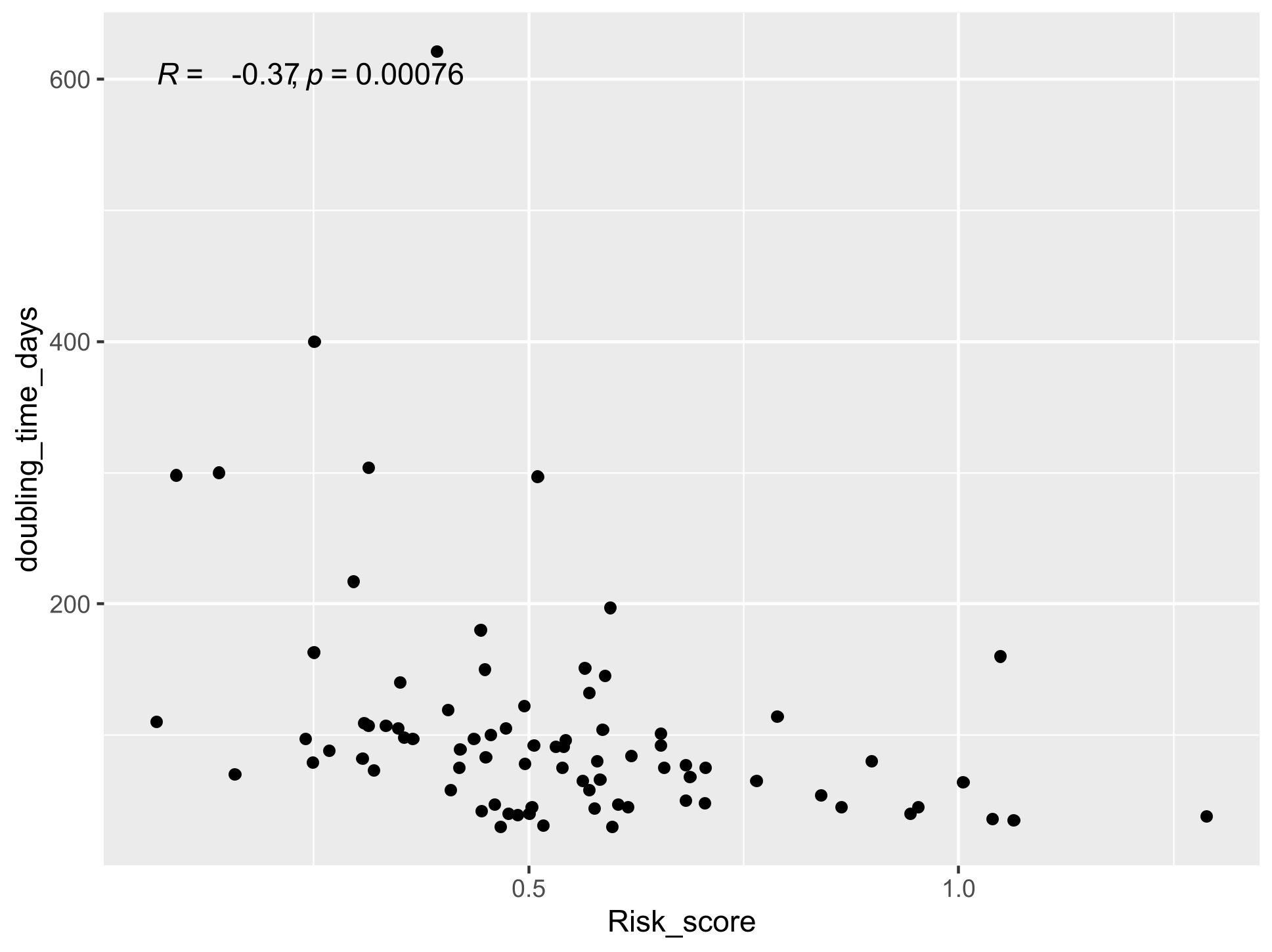

Supplement: Supplementary file 4 [file Image4.JPEG]

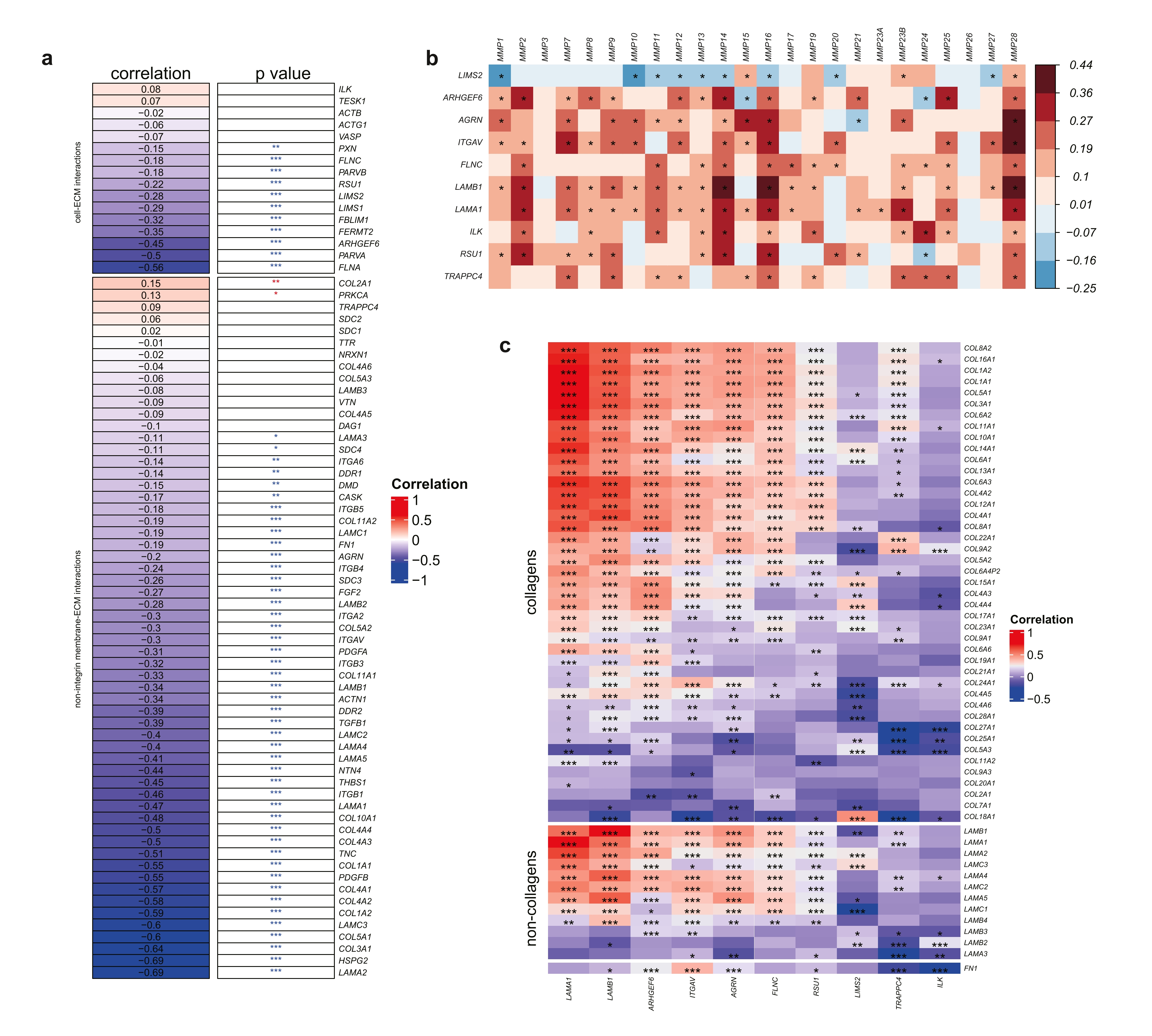

Supplement: Supplementary file 5 [file Image2.JPEG]
