# Supplementary material for: Stemness analysis in hepatocellular carcinoma identifies an extracellular matrix gene–related signature associated with prognosis and therapy response
Source: Front Genet. 2022 Aug 30;13:959834. doi: 10.3389/fgene.2022.959834 (PMC9468756; doi:10.3389/fgene.2022.959834)
Supplement: Supplementary file 2 [file Table1.DOCX]

**Supplemental Table 1** The mRNAsi and EREG-mRNAsi of TCGA-HCC patients.

| **Sample ID** | **mRNAsi** | **EREG-mRNAsi** |
| --- | --- | --- |
| TCGA-2V-A95S-01 | 0.402031 | 0.627018 |
| TCGA-2Y-A9GS-01 | 0.442297 | 0.618124 |
| TCGA-2Y-A9GT-01 | 0.300768 | 0.515315 |
| TCGA-2Y-A9GU-01 | 0.375268 | 0.40588 |
| TCGA-2Y-A9GV-01 | 0.310258 | 0.551925 |
| TCGA-2Y-A9GW-01 | 0.325595 | 0.626503 |
| TCGA-2Y-A9GX-01 | 0.256915 | 0.585544 |
| TCGA-2Y-A9GY-01 | 0.467311 | 0.721172 |
| TCGA-2Y-A9GZ-01 | 0.398377 | 0.630855 |
| TCGA-2Y-A9H0-01 | 0.512639 | 0.521223 |
| TCGA-2Y-A9H1-01 | 0.428315 | 0.638065 |
| TCGA-2Y-A9H2-01 | 0.373168 | 0.485375 |
| TCGA-2Y-A9H3-01 | 0.343728 | 0.474759 |
| TCGA-2Y-A9H4-01 | 0.444588 | 0.718201 |
| TCGA-2Y-A9H5-01 | 0.302689 | 0.589496 |
| TCGA-2Y-A9H6-01 | 0.272553 | 0.473183 |
| TCGA-2Y-A9H7-01 | 0.415275 | 0.527567 |
| TCGA-2Y-A9H8-01 | 0.434945 | 0.475067 |
| TCGA-2Y-A9H9-01 | 0.390973 | 0.497109 |
| TCGA-2Y-A9HA-01 | 0.410491 | 0.585048 |
| TCGA-2Y-A9HB-01 | 0.378926 | 0.525902 |
| TCGA-3K-AAZ8-01 | 0.421625 | 0.688997 |
| TCGA-4R-AA8I-01 | 0.394801 | 0.481699 |
| TCGA-5C-A9VG-01 | 0.421896 | 0.607537 |
| TCGA-5C-A9VH-01 | 0.301258 | 0.566114 |
| TCGA-5R-AA1C-01 | 0.422825 | 0.693002 |
| TCGA-5R-AA1D-01 | 0.214527 | 0.721845 |
| TCGA-5R-AAAM-01 | 0.27347 | 0.731396 |
| TCGA-BC-4072-01 | | 0.705387 |
| TCGA-BC-4073-01 | 0.378525 | 0.711994 |
| TCGA-BC-A10Q-01 | 0.301564 | 0.427818 |
| TCGA-BC-A10R-01 | 0.288976 | 0.492539 |
| TCGA-BC-A10S-01 | | 0.594824 |
| TCGA-BC-A10T-01 | 0.359388 | 0.744334 |
| TCGA-BC-A10U-01 | 0.381205 | 0.57927 |
| TCGA-BC-A10W-01 | 0.459213 | 0.641636 |
| TCGA-BC-A10X-01 | 0.279073 | 0.565045 |
| TCGA-BC-A10Y-01 | 0.46229 | 0.610791 |
| TCGA-BC-A10Z-01 | 0.466465 | 0.596177 |
| TCGA-BC-A110-01 | | 0.620483 |
| TCGA-BC-A112-01 | | 0.611492 |
| TCGA-BC-A216-01 | 0.417796 | 0.547873 |
| TCGA-BC-A217-01 | 0.472464 | 0.708111 |
| TCGA-BC-A3KF-01 | 0.402669 | 0.529726 |
| TCGA-BC-A3KG-01 | | 0.591014 |
| TCGA-BC-A5W4-01 | 0.441269 | 0.656513 |
| TCGA-BC-A69H-01 | 0.462116 | 0.817416 |
| TCGA-BC-A69I-01 | | 0.641513 |
| TCGA-BC-A8YO-01 | 0.450591 | 0.536462 |
| TCGA-BD-A2L6-01 | 0.422291 | 0.605823 |
| TCGA-BD-A3EP-01 | 0.292603 | 0.610118 |
| TCGA-BW-A5NO-01 | 0.426323 | 0.652451 |
| TCGA-BW-A5NP-01 | 0.423895 | 0.606498 |
| TCGA-BW-A5NQ-01 | 0.538779 | 0.704903 |
| TCGA-CC-5258-01 | 0.444544 | 0.683573 |
| TCGA-CC-5259-01 | 0.418499 | 0.645054 |
| TCGA-CC-5260-01 | 0.305092 | 0.557459 |
| TCGA-CC-5261-01 | | 0.616145 |
| TCGA-CC-5262-01 | 0.355584 | 0.749291 |
| TCGA-CC-5263-01 | 0.412725 | 0.5922 |
| TCGA-CC-5264-01 | 0.455408 | 0.542785 |
| TCGA-CC-A123-01 | 0.457049 | 0.718974 |
| TCGA-CC-A1HT-01 | 0.398298 | 0.698117 |
| TCGA-CC-A3M9-01 | 0.39195 | 0.586436 |
| TCGA-CC-A3MA-01 | 0.325286 | 0.73494 |
| TCGA-CC-A3MB-01 | 0.408021 | 0.607595 |
| TCGA-CC-A3MC-01 | 0.405649 | 0.625826 |
| TCGA-CC-A5UC-01 | 0.426954 | 0.693412 |
| TCGA-CC-A5UD-01 | 0.359149 | 0.776962 |
| TCGA-CC-A5UE-01 | 0.468795 | 0.64143 |
| TCGA-CC-A7IE-01 | 0.417867 | 0.568657 |
| TCGA-CC-A7IF-01 | 0.400888 | 0.583016 |
| TCGA-CC-A7IG-01 | 0.422419 | 0.668839 |
| TCGA-CC-A7IH-01 | 0.330009 | 0.675253 |
| TCGA-CC-A7II-01 | 0.520586 | 0.69808 |
| TCGA-CC-A7IJ-01 | 0.343349 | 0.581926 |
| TCGA-CC-A7IK-01 | 0.428169 | 0.593716 |
| TCGA-CC-A7IL-01 | 0.461129 | 0.78624 |
| TCGA-CC-A8HT-01 | 0.497121 | 0.666488 |
| TCGA-CC-A8HU-01 | 0.452672 | 0.479385 |
| TCGA-CC-A8HV-01 | 0.393709 | 0.606775 |
| TCGA-CC-A9FS-01 | 0.422264 | 0.507298 |
| TCGA-CC-A9FU-01 | 0.47313 | 0.602531 |
| TCGA-CC-A9FV-01 | 0.19565 | 0.628996 |
| TCGA-CC-A9FW-01 | 0.381859 | 0.576404 |
| TCGA-DD-A113-01 | 0.405739 | 0.557253 |
| TCGA-DD-A114-01 | 0.361394 | 0.506898 |
| TCGA-DD-A115-01 | 0.376121 | 0.488718 |
| TCGA-DD-A116-01 | 0.384494 | 0.64354 |
| TCGA-DD-A118-01 | 0.374641 | 0.515532 |
| TCGA-DD-A119-01 | 0.39306 | 0.608984 |
| TCGA-DD-A11A-01 | 0.426665 | 0.528701 |
| TCGA-DD-A11B-01 | 0.380867 | 0.54568 |
| TCGA-DD-A11C-01 | 0.386527 | 0.549391 |
| TCGA-DD-A11D-01 | 0.328946 | 0.60102 |
| TCGA-DD-A1EA-01 | | 0.659767 |
| TCGA-DD-A1EB-01 | 0.373463 | 0.573628 |
| TCGA-DD-A1EC-01 | 0.380408 | 0.540299 |
| TCGA-DD-A1ED-01 | 0.27678 | 0.559567 |
| TCGA-DD-A1EE-01 | 0.40926 | 0.616605 |
| TCGA-DD-A1EF-01 | 0.37494 | 0.478925 |
| TCGA-DD-A1EG-01 | | 0.691169 |
| TCGA-DD-A1EH-01 | 0.36533 | 0.529474 |
| TCGA-DD-A1EI-01 | 0.310475 | 0.618631 |
| TCGA-DD-A1EJ-01 | 0.421237 | 0.60287 |
| TCGA-DD-A1EK-01 | 0.359058 | 0.586784 |
| TCGA-DD-A1EL-01 | 0.458986 | 0.808061 |
| TCGA-DD-A39V-01 | 0.412735 | 0.692203 |
| TCGA-DD-A39W-01 | 0.361751 | 0.670813 |
| TCGA-DD-A39X-01 | 0.420783 | 0.682803 |
| TCGA-DD-A39Y-01 | | 0.678218 |
| TCGA-DD-A39Z-01 | 0.435936 | 0.608534 |
| TCGA-DD-A3A1-01 | | 0.675445 |
| TCGA-DD-A3A2-01 | 0.378895 | 0.547947 |
| TCGA-DD-A3A3-01 | 0.442435 | 0.684215 |
| TCGA-DD-A3A4-01 | 0.427228 | 0.73378 |
| TCGA-DD-A3A5-01 | 0.40667 | 0.715284 |
| TCGA-DD-A3A6-01 | 0.211989 | 0.536742 |
| TCGA-DD-A3A7-01 | 0.451231 | 0.570872 |
| TCGA-DD-A3A8-01 | 0.354039 | 0.643215 |
| TCGA-DD-A3A9-01 | 0.316246 | 0.559871 |
| TCGA-DD-A4NA-01 | 0.300082 | 0.518137 |
| TCGA-DD-A4ND-01 | 0.229759 | 0.504921 |
| TCGA-DD-A4NE-01 | 0.38901 | 0.524692 |
| TCGA-DD-A4NF-01 | 0.326269 | 0.569855 |
| TCGA-DD-A4NG-01 | 0.359498 | 0.642803 |
| TCGA-DD-A4NH-01 | 0.343797 | 0.56016 |
| TCGA-DD-A4NI-01 | 0.30464 | 0.575653 |
| TCGA-DD-A4NJ-01 | 0.389383 | 0.514085 |
| TCGA-DD-A4NK-01 | 0.366395 | 0.536703 |
| TCGA-DD-A4NL-01 | 0.276102 | 0.607002 |
| TCGA-DD-A4NN-01 | 0.38072 | 0.582685 |
| TCGA-DD-A4NO-01 | 0.34185 | 0.590244 |
| TCGA-DD-A4NP-01 | 0.328091 | 0.592813 |
| TCGA-DD-A4NQ-01 | 0.425754 | 0.636061 |
| TCGA-DD-A4NR-01 | 0.403023 | 0.662447 |
| TCGA-DD-A4NS-01 | 0.16707 | 0.536502 |
| TCGA-DD-A4NV-01 | 0.291854 | 0.59356 |
| TCGA-DD-A73A-01 | 0.387149 | 0.646046 |
| TCGA-DD-A73B-01 | 0.464943 | 0.553956 |
| TCGA-DD-A73C-01 | 0.354957 | 0.504669 |
| TCGA-DD-A73D-01 | 0.463902 | 0.687066 |
| TCGA-DD-A73E-01 | 0.350253 | 0.595382 |
| TCGA-DD-A73F-01 | 0.429181 | 0.640756 |
| TCGA-DD-A73G-01 | 0.417478 | 0.626112 |
| TCGA-DD-AAC8-01 | 0.369215 | 0.684997 |
| TCGA-DD-AAC9-01 | 0.336896 | 0.719177 |
| TCGA-DD-AACA-01 | 0.366491 | 0.679449 |
| TCGA-DD-AACB-01 | 0.416732 | 0.724028 |
| TCGA-DD-AACD-01 | 0.434478 | 0.676864 |
| TCGA-DD-AACE-01 | 0.369165 | 0.558936 |
| TCGA-DD-AACF-01 | 0.459436 | 0.540684 |
| TCGA-DD-AACG-01 | 0.526861 | 0.702585 |
| TCGA-DD-AACH-01 | 0.392067 | 0.517656 |
| TCGA-DD-AACI-01 | 0.399266 | 0.596836 |
| TCGA-DD-AACJ-01 | 0.455669 | 0.639425 |
| TCGA-DD-AACK-01 | 0.413389 | 0.665305 |
| TCGA-DD-AACL-01 | 0.478641 | 0.633465 |
| TCGA-DD-AACN-01 | 0.292954 | 0.56801 |
| TCGA-DD-AACO-01 | 0.43894 | 0.567253 |
| TCGA-DD-AACP-01 | 0.475997 | 0.663798 |
| TCGA-DD-AACQ-01 | 0.407148 | 0.474946 |
| TCGA-DD-AACS-01 | 0.423783 | 0.555468 |
| TCGA-DD-AACT-01 | 0.346283 | 0.722242 |
| TCGA-DD-AACU-01 | 0.358327 | 0.667004 |
| TCGA-DD-AACV-01 | 0.496065 | 0.539598 |
| TCGA-DD-AACW-01 | 0.439321 | 0.707808 |
| TCGA-DD-AACX-01 | 0.438483 | 0.601517 |
| TCGA-DD-AACY-01 | 0.375079 | 0.560695 |
| TCGA-DD-AACZ-01 | 0.456029 | 0.66803 |
| TCGA-DD-AAD0-01 | 0.415582 | 0.630225 |
| TCGA-DD-AAD1-01 | 0.252927 | 0.599361 |
| TCGA-DD-AAD2-01 | 0.317213 | 0.639694 |
| TCGA-DD-AAD3-01 | 0.271082 | 0.634957 |
| TCGA-DD-AAD5-01 | 0.415939 | 0.708823 |
| TCGA-DD-AAD6-01 | 0.486117 | 0.526106 |
| TCGA-DD-AAD8-01 | 0.410428 | 0.541418 |
| TCGA-DD-AADA-01 | 0.309995 | 0.537588 |
| TCGA-DD-AADB-01 | 0.43594 | 0.665957 |
| TCGA-DD-AADC-01 | 0.441301 | 0.698526 |
| TCGA-DD-AADD-01 | 0.482728 | 0.595669 |
| TCGA-DD-AADF-01 | 0.480084 | 0.557828 |
| TCGA-DD-AADG-01 | 0.370285 | 0.709474 |
| TCGA-DD-AADI-01 | 0.351243 | 0.595041 |
| TCGA-DD-AADJ-01 | 0.39022 | 0.648143 |
| TCGA-DD-AADK-01 | 0.341677 | 0.58829 |
| TCGA-DD-AADL-01 | 0.420094 | 0.627347 |
| TCGA-DD-AADM-01 | 0.443701 | 0.723691 |
| TCGA-DD-AADN-01 | 0.564323 | 0.764681 |
| TCGA-DD-AADO-01 | 0.471166 | 0.712427 |
| TCGA-DD-AADP-01 | 0.366348 | 0.469605 |
| TCGA-DD-AADQ-01 | 0.445426 | 0.615308 |
| TCGA-DD-AADR-01 | 0.495597 | 0.70242 |
| TCGA-DD-AADS-01 | 0.438866 | 0.676328 |
| TCGA-DD-AADU-01 | 0.380491 | 0.639485 |
| TCGA-DD-AADV-01 | 0.383103 | 0.538913 |
| TCGA-DD-AADW-01 | 0.340947 | 0.482302 |
| TCGA-DD-AADY-01 | 0.397051 | 0.557781 |
| TCGA-DD-AAE0-01 | 0.392651 | 0.622018 |
| TCGA-DD-AAE1-01 | 0.412693 | 0.584232 |
| TCGA-DD-AAE2-01 | 0.387492 | 0.496287 |
| TCGA-DD-AAE3-01 | 0.354169 | 0.734094 |
| TCGA-DD-AAE4-01 | 0.441972 | 0.709927 |
| TCGA-DD-AAE6-01 | 0.514441 | 0.514347 |
| TCGA-DD-AAE7-01 | 0.31036 | 0.688587 |
| TCGA-DD-AAE9-01 | 0.397604 | 0.642454 |
| TCGA-DD-AAEA-01 | 0.440954 | 0.591145 |
| TCGA-DD-AAEB-01 | 0.367846 | 0.645754 |
| TCGA-DD-AAED-01 | 0.420747 | 0.525383 |
| TCGA-DD-AAEE-01 | 0.400954 | 0.582501 |
| TCGA-DD-AAEG-01 | 0.399662 | 0.656322 |
| TCGA-DD-AAEH-01 | 0.393716 | 0.595417 |
| TCGA-DD-AAEI-01 | 0.463446 | 0.657705 |
| TCGA-DD-AAEK-01 | 0.325497 | 0.5986 |
| TCGA-DD-AAVP-01 | 0.374294 | 0.580822 |
| TCGA-DD-AAVQ-01 | 0.376291 | 0.667562 |
| TCGA-DD-AAVR-01 | 0.320553 | 0.615361 |
| TCGA-DD-AAVS-01 | 0.375799 | 0.697052 |
| TCGA-DD-AAVU-01 | 0.48823 | 0.681063 |
| TCGA-DD-AAVV-01 | 0.328085 | 0.6467 |
| TCGA-DD-AAVW-01 | 0.272509 | 0.589751 |
| TCGA-DD-AAVX-01 | 0.383138 | 0.657738 |
| TCGA-DD-AAVY-01 | 0.421612 | 0.669944 |
| TCGA-DD-AAVZ-01 | 0.404743 | 0.65051 |
| TCGA-DD-AAW0-01 | 0.323632 | 0.642756 |
| TCGA-DD-AAW1-01 | 0.348154 | 0.578448 |
| TCGA-DD-AAW2-01 | 0.379846 | 0.615862 |
| TCGA-DD-AAW3-01 | 0.394061 | 0.604687 |
| TCGA-ED-A459-01 | 0.420213 | 0.550021 |
| TCGA-ED-A4XI-01 | 0.267619 | 0.589172 |
| TCGA-ED-A5KG-01 | 0.322491 | 0.570278 |
| TCGA-ED-A627-01 | 0.20824 | 0.56004 |
| TCGA-ED-A66X-01 | 0.344244 | 0.674807 |
| TCGA-ED-A66Y-01 | 0.35678 | 0.461174 |
| TCGA-ED-A7PX-01 | 0.348076 | 0.675495 |
| TCGA-ED-A7PY-01 | 0.385131 | 0.600201 |
| TCGA-ED-A7PZ-01 | 0.438983 | 0.653406 |
| TCGA-ED-A7XO-01 | 0.377698 | 0.539876 |
| TCGA-ED-A7XP-01 | 0.352234 | 0.621656 |
| TCGA-ED-A82E-01 | 0.289304 | 0.631072 |
| TCGA-ED-A8O5-01 | 0.364399 | 0.514157 |
| TCGA-ED-A8O6-01 | 0.403047 | 0.636233 |
| TCGA-EP-A12J-01 | 0.378369 | 0.597646 |
| TCGA-EP-A26S-01 | 0.377926 | 0.662252 |
| TCGA-EP-A2KA-01 | 0.406398 | 0.729825 |
| TCGA-EP-A2KB-01 | 0.382002 | 0.56931 |
| TCGA-EP-A2KC-01 | 0.352516 | 0.620474 |
| TCGA-EP-A3JL-01 | 0.39525 | 0.639419 |
| TCGA-EP-A3RK-01 | 0.417864 | 0.687722 |
| TCGA-ES-A2HS-01 | 0.370901 | 0.57308 |
| TCGA-ES-A2HT-01 | 0.387176 | 0.696994 |
| TCGA-FV-A23B-01 | 0.373811 | 0.544181 |
| TCGA-FV-A2QQ-01 | 0.401455 | 0.674131 |
| TCGA-FV-A2QR-01 | 0.322481 | 0.671512 |
| TCGA-FV-A3I1-01 | 0.371489 | 0.611907 |
| TCGA-FV-A3R2-01 | 0.434428 | 0.650305 |
| TCGA-FV-A3R3-01 | 0.228859 | 0.647363 |
| TCGA-FV-A495-01 | 0.380867 | 0.593308 |
| TCGA-FV-A496-01 | 0.375097 | 0.519196 |
| TCGA-FV-A4ZP-01 | 0.480009 | 0.826216 |
| TCGA-FV-A4ZQ-01 | 0.463726 | 0.666089 |
| TCGA-G3-A25S-01 | 0.497043 | 0.62029 |
| TCGA-G3-A25T-01 | 0.334061 | 0.450455 |
| TCGA-G3-A25U-01 | 0.467122 | 0.708159 |
| TCGA-G3-A25V-01 | 0.306916 | 0.575341 |
| TCGA-G3-A25X-01 | | 0.646277 |
| TCGA-G3-A25Y-01 | 0.336551 | 0.701083 |
| TCGA-G3-A25Z-01 | 0.39426 | 0.699295 |
| TCGA-G3-A3CH-01 | 0.372589 | 0.628205 |
| TCGA-G3-A3CI-01 | 0.316849 | 0.68192 |
| TCGA-G3-A3CJ-01 | | 0.701896 |
| TCGA-G3-A3CK-01 | 0.390696 | 0.616701 |
| TCGA-G3-A5SI-01 | 0.465125 | 0.508926 |
| TCGA-G3-A5SJ-01 | 0.339752 | 0.570046 |
| TCGA-G3-A5SK-01 | 0.33712 | 0.428232 |
| TCGA-G3-A5SL-01 | 0.363978 | 0.711655 |
| TCGA-G3-A5SM-01 | 0.341561 | 0.558942 |
| TCGA-G3-A6UC-01 | 0.398493 | 0.651809 |
| TCGA-G3-A7M5-01 | 0.445357 | 0.634396 |
| TCGA-G3-A7M6-01 | 0.270556 | 0.492673 |
| TCGA-G3-A7M7-01 | 0.387499 | 0.697945 |
| TCGA-G3-A7M8-01 | 0.337297 | 0.609524 |
| TCGA-G3-A7M9-01 | 0.465847 | 0.598513 |
| TCGA-G3-AAUZ-01 | 0.412786 | 0.650547 |
| TCGA-G3-AAV0-01 | 0.374233 | 0.469544 |
| TCGA-G3-AAV1-01 | 0.389032 | 0.641201 |
| TCGA-G3-AAV2-01 | 0.34308 | 0.539451 |
| TCGA-G3-AAV3-01 | 0.357437 | 0.596443 |
| TCGA-G3-AAV4-01 | 0.392326 | 0.646043 |
| TCGA-G3-AAV5-01 | 0.456685 | 0.715286 |
| TCGA-G3-AAV6-01 | 0.402073 | 0.628586 |
| TCGA-G3-AAV7-01 | 0.409434 | 0.636403 |
| TCGA-GJ-A3OU-01 | 0.292244 | 0.66006 |
| TCGA-GJ-A6C0-01 | 0.366664 | 0.557398 |
| TCGA-GJ-A9DB-01 | 0.329618 | 0.568613 |
| TCGA-HP-A5MZ-01 | 0.270256 | 0.603775 |
| TCGA-HP-A5N0-01 | 0.276217 | 0.613346 |
| TCGA-K7-A5RF-01 | 0.278351 | 0.638476 |
| TCGA-K7-A5RG-01 | 0.400379 | 0.497011 |
| TCGA-K7-A6G5-01 | 0.356875 | 0.683115 |
| TCGA-KR-A7K0-01 | 0.299156 | 0.488858 |
| TCGA-KR-A7K7-01 | 0.480736 | 0.627404 |
| TCGA-KR-A7K8-01 | 0.356515 | 0.678108 |
| TCGA-LG-A6GG-01 | 0.374384 | 0.554233 |
| TCGA-LG-A9QC-01 | 0.34927 | 0.718598 |
| TCGA-LG-A9QD-01 | 0.352649 | 0.569272 |
| TCGA-MI-A75C-01 | 0.452562 | 0.665511 |
| TCGA-MI-A75E-01 | 0.34176 | 0.684467 |
| TCGA-MI-A75G-01 | 0.397396 | 0.567861 |
| TCGA-MI-A75H-01 | 0.378784 | 0.672803 |
| TCGA-MI-A75I-01 | 0.42521 | 0.613705 |
| TCGA-MR-A520-01 | 0.360867 | 0.665449 |
| TCGA-NI-A4U2-01 | 0.34791 | 0.543651 |
| TCGA-NI-A8LF-01 | 0.280371 | 0.628951 |
| TCGA-O8-A75V-01 | 0.361047 | 0.649341 |
| TCGA-PD-A5DF-01 | 0.330035 | 0.65806 |
| TCGA-QA-A7B7-01 | 0.490004 | 0.592619 |
| TCGA-RC-A6M3-01 | 0.48241 | 0.673587 |
| TCGA-RC-A6M4-01 | 0.426914 | 0.646315 |
| TCGA-RC-A6M6-01 | 0.420896 | 0.947825 |
| TCGA-RC-A7S9-01 | 0.438522 | 0.393851 |
| TCGA-RC-A7SB-01 | 0.383447 | 0.739378 |
| TCGA-RC-A7SF-01 | 0.363764 | 0.508127 |
| TCGA-RC-A7SH-01 | 0.407888 | 0.511191 |
| TCGA-RC-A7SK-01 | 0.39048 | 0.786995 |
| TCGA-RG-A7D4-01 | 0.494867 | 0.61414 |
| TCGA-UB-A7MB-01 | 0.451733 | 0.550876 |
| TCGA-UB-A7MC-01 | 0.432233 | 0.501197 |
| TCGA-UB-A7MD-01 | 0.338971 | 0.630326 |
| TCGA-UB-A7ME-01 | 0.36971 | 0.556302 |
| TCGA-UB-A7MF-01 | 0.433116 | 0.471944 |
| TCGA-UB-AA0U-01 | 0.342683 | 0.541265 |
| TCGA-UB-AA0V-01 | 0.268809 | 0.552588 |
| TCGA-WJ-A86L-01 | 0.384843 | 0.567935 |
| TCGA-WQ-A9G7-01 | 0.433125 | 0.488991 |
| TCGA-WQ-AB4B-01 | 0.364401 | 0.680557 |
| TCGA-WX-AA44-01 | 0.345778 | 0.521092 |
| TCGA-WX-AA46-01 | 0.331201 | 0.519428 |
| TCGA-WX-AA47-01 | 0.423842 | 0.47727 |
| TCGA-XR-A8TD-01 | 0.340037 | 0.748132 |
| TCGA-XR-A8TE-01 | 0.27557 | 0.663944 |
| TCGA-XR-A8TF-01 | 0.447786 | 0.559749 |
| TCGA-XR-A8TG-01 | 0.337954 | 0.54024 |
| TCGA-YA-A8S7-01 | 0.305094 | 0.611926 |
| TCGA-ZP-A9CV-01 | 0.369955 | 0.534825 |
| TCGA-ZP-A9CY-01 | 0.306329 | 0.562849 |
| TCGA-ZP-A9CZ-01 | 0.431143 | 0.551008 |
| TCGA-ZP-A9D0-01 | 0.372313 | 0.43876 |
| TCGA-ZP-A9D1-01 | 0.315447 | 0.652754 |
| TCGA-ZP-A9D2-01 | 0.325387 | 0.568131 |
| TCGA-ZP-A9D4-01 | 0.396879 | 0.556434 |
| TCGA-ZS-A9CD-01 | 0.326131 | 0.622314 |
| TCGA-ZS-A9CE-01 | 0.421236 | 0.704698 |
| TCGA-ZS-A9CF-01 | 0.394743 | 0.588833 |
| TCGA-ZS-A9CG-01 | 0.301896 | 0.729936 |
