# Supplementary material for: Stemness analysis in hepatocellular carcinoma identifies an extracellular matrix gene–related signature associated with prognosis and therapy response
Source: Front Genet. 2022 Aug 30;13:959834. doi: 10.3389/fgene.2022.959834 (PMC9468756; doi:10.3389/fgene.2022.959834)
Supplement: Supplementary file 6 [file Table2.DOCX]

**Supplemental Table 2** 75 ECM-related genes used for LASSO regression analysis.

| pathway | Gene |
| --- | --- |
| cell-ECM interactions | ILK |
|  | TESK1 |
|  | ACTB |
|  | ACTG1 |
|  | VASP |
|  | PXN |
|  | FLNC |
|  | PARVB |
|  | RSU1 |
|  | LIMS2 |
|  | LIMS1 |
|  | FBLIM1 |
|  | FERMT2 |
|  | ARHGEF6 |
|  | PARVA |
|  | FLNA |
| Non-integrin membrane-ECM interactions | COL2A1 |
|  | PRKCA |
|  | TRAPPC4 |
|  | SDC2 |
|  | SDC1 |
|  | TTR |
|  | NRXN1 |
|  | COL4A6 |
|  | COL5A3 |
|  | LAMB3 |
|  | VTN |
|  | COL4A5 |
|  | DAG1 |
|  | LAMA3 |
|  | SDC4 |
|  | ITGA6 |
|  | DDR1 |
|  | DMD |
|  | CASK |
|  | ITGB5 |
|  | COL11A2 |
|  | LAMC1 |
|  | FN1 |
|  | AGRN |
|  | ITGB4 |
|  | SDC3 |
|  | FGF2 |
|  | LAMB2 |
|  | ITGA2 |
|  | COL5A2 |
|  | ITGAV |
|  | PDGFA |
|  | ITGB3 |
|  | COL11A1 |
|  | LAMB1 |
|  | ACTN1 |
|  | DDR2 |
|  | TGFB1 |
|  | LAMC2 |
|  | LAMA4 |
|  | LAMA5 |
|  | NTN4 |
|  | THBS1 |
|  | ITGB1 |
|  | LAMA1 |
|  | COL10A1 |
|  | COL4A4 |
|  | COL4A3 |
|  | TNC |
|  | COL1A1 |
|  | PDGFB |
|  | COL4A1 |
|  | COL4A2 |
|  | COL1A2 |
|  | LAMC3 |
|  | COL5A1 |
|  | COL3A1 |
|  | HSPG2 |
|  | LAMA2 |
